# Brainwide Analysis of Functional Connectivity Patterns in Specific Phobia and Its Treatment

**DOI:** 10.1016/j.bpsgos.2025.100562

**Published:** 2025-07-08

**Authors:** Markus Muehlhan, Judith Schäfer, Kevin Hilbert, Esther Seidl, Katja Beesdo-Baum

**Affiliations:** a Department of Psychology, Faculty of Human Sciences, MSH Medical School Hamburg, Hamburg, Germany; b ICAN Institute of Cognitive and Affective Neuroscience, MSH Medical School Hamburg, Hamburg, Germany; c Behavioral Epidemiology, Institute of Clinical Psychology and Psychotherapy, Dresden University of Technology, Dresden, Germany; d Department of Psychology, HMU Health and Medical University Erfurt, Erfurt, Germany

**Keywords:** Connectivity, fc-MVPA, fMRI, One-session treatment, Resting-state, Specific phobia

## Abstract

**Background:**

Specific phobia (SP) is a prevalent mental disorder for which exposure-based treatments are the most effective. Little is known about the intrinsic functional connectivity of SP and its modification by treatment. While previous studies were limited to a priori–defined brain regions, we used connectome-wide analyses to capture the full extent of altered functional connectivity.

**Methods:**

We used functional magnetic resonance imaging in combination with hypothesis-free, data-driven functional connectivity multivariate pattern analysis (fc-MVPA) to identify differences between 72 individuals with SP and a nonphobic control group (CG) (*n* = 82). The SP group then received a one-session exposure treatment and was scanned again 9 weeks later on average.

**Results:**

fc-MVPA identified the largest differences between the SP group and CG in sensorimotor regions, with lower connectivity to temporal nodes of the default network and anticorrelations with the frontoparietal network in the SP group compared with the CG. Stronger connectivity in the pretreatment compared with the posttreatment condition was evident in visual regions, while anticorrelations with the frontoparietal network were reduced. Post hoc comparisons showed that the connectivity strengths of the SP group after treatment between almost all identified nodes of both contrasts (SP vs. CG and pre vs. post) were comparable to those of the CG at baseline.

**Conclusions:**

Given the known functions of the identified networks, it is possible that the changes in connectivity measured after treatment indicate improved action control, enabled by more accurate prediction of the environment and stronger coupling of perceptual and action regions with higher-order control regions.

## Plain Language Summary

Specific phobia (SP) is a prevalent mental disorder, but little is known about the intrinsic functional connectivity of SP and its modification by treatment. We used functional magnetic resonance imaging combined with data-driven analysis to answer these questions: where in the brain does connectivity differ between individuals with SP and control individuals and where in the brain does connectivity differ before and after exposure treatment? Resulting clusters were identified in regions of the sensorimotor, default, visual, and frontoparietal networks, whose interaction changed after treatment, possibly indicating improved cognitive-affective control of perceptual and action regions.

Specific phobia (SP) is an intense and irrational fear that may be associated with objects or situations that pose little or no actual danger (1). It is one of the most common mental disorders, with a median lifetime prevalence of 7.2% across several countries (2). Although adults with SP can recognize that the fear is exaggerated or unfounded, even the thought of the feared object or situation triggers severe anxiety symptoms and can lead to impairment in several areas of daily life, such as home management, ability to work, and social life (3). Exposure-based treatments are the most effective therapy for SP. The phobic person is therapeutically guided toward the phobic object with the aim of learning that the expected feared outcome does not occur, which usually results in a reduction of the fear (4). While the effectiveness of these therapies is well documented (5), the neural basis of SP and its treatment is relatively unclear. Most functional neuroimaging studies have been task based, using phobic stimuli or fear condition paradigms, for example, to investigate changes in activation in associated brain structures. These studies identified several brain regions that are critical for processing fear, anxiety, and emotional responses such as the amygdalae or parts of the anterior cingulate cortices, the insulae, or hippocampal regions [e.g., (6, 7, 8, 9)]. While these studies have provided important insights into the neurobiology of SP, they have not been able to explain the disorder to the extent that a comprehensive neurobiological model could be developed (10). One reason for this is the strong focus on threat or emotion processing, which localizes only the brain regions involved in processing these stimuli but not those involved in other processes that may also be relevant to the disorder. Another is that localization studies can only provide limited information about brain organization. Connectivity analyses, which measure functional integration, are better at explaining behavior, cognition, and emotion because they describe how different brain regions work together (11). However, when functional connectivity is measured in the context of a specific task, the focus remains on networks associated with the task. A promising approach to overcome this problem is task-independent resting-state analysis, in which intrinsic connectivity is measured. This can circumvent some of these interpretative ambiguities and may allow the identification of more fundamental deviations that underlie the disorders (12). In addition, if no tasks are used, there can be no learning effects that could bias the results of the pre- and postmeasurements (12). Initial resting-state analyses based on regions of interest (ROIs) derived from previous stimulation studies yielded heterogeneous results. One study found no difference between a SP group and a control group (CG) (13). Another study was able to predict the response to treatment based on a pretreatment measurement, but this could not be replicated in another sample (14). The selection of predefined ROIs used as seed regions has the advantage that the analyses are relatively easy to perform due to the reduction of analysis units compared with a voxel-to-voxel analysis of the whole brain (15). Ultimately, however, only the functional connectivity of the respective seed region is measured, which carries the risk of potentially false negative results, as any effects that are not in the area of the seed connectivity will be missed. Even when intrinsic networks are calculated through independent component analysis (ICA), specific networks are selected for investigation, and anything outside these networks is not taken into account.

The problem of spatial restrictions can be surmounted by utilizing brainwide connectome inferences. However, such inferences are susceptible to high dimensionality, as each connectivity between voxels of gray matter is computed. One solution to this problem is functional connectivity multivariate pattern analysis (fc-MVPA), which uses dimensionality reduction to characterize the heterogeneity of functional connectivity patterns between a voxel and the rest of the brain and to identify regions with differences between patients and control participants or between pre- and posttreatment conditions (15).

The objective of the current study was to analyze functional magnetic resonance imaging (fMRI) resting-state data using an fc-MVPA. This hypothesis-free, data-driven, connectome-wide approach has the potential to provide new insights into functional connectivity alterations in SP and offer new starting points for future research. Therefore, individuals diagnosed with one of the most common types of SP, spider phobia (16,17), formed the intervention group, which was compared with a nonphobic CG. Group differences were first estimated in a cross-sectional case-control comparison of resting-state data. The spider phobia group then received a single-session exposure therapy and were scanned again to identify pre- versus posttreatment differences in functional connectivity.

## Methods and Materials

All information on the methods is briefly presented here. Detailed information can be found in the Supplement.

### Participants

Participants were recruited from the general population using flyers and advertisements and from collaborating clinical institutions such as the institutes’ outpatient psychotherapy centers. A total of 72 individuals (age 25.5 [6.8] years; 91.7% female) with primary DSM-5 defined specific phobia—animal (spider) subtype (1)—and 82 comparison participants (age 23.8 [5.1]; 90.2% female) without any lifetime or current mental disorder formed the main analysis sample.

Sociodemographic and clinical data are detailed in Table S1. All participants were of European descent and reported no neurological or endocrinological disorders. The study protocol was reviewed and accepted by the Ethics Committee of the Dresden University of Technology (EK 543122015).

### Procedure

Participants were invited to a baseline assessment, which included the acquisition of functional and structural MR images. About 6 weeks later (mean [SD] = 5.74 [4.26]), individuals with SP received single-session exposure-based therapy as proposed by Öst (18). This treatment intervention incorporates techniques from the full spectrum of cognitive behavioral therapy but relies primarily on exposure and is limited to a single 3-hour session. The SP group underwent a second (f)MRI scan approximately 15 weeks (mean [SD] = 15.13 [7.83]) after the baseline measurement and approximately 9 weeks after the treatment (mean [SD] = 9.37 [4.78]). The CG only participated at baseline. A sensitivity analysis of the 10 participants who dropped out of the study after the baseline measurement is available in the Supplement.

### Image Acquisition and Analyses

Images were acquired using a 3T whole-body scanner with a 32-channel head coil. A total of 450 whole-brain volumes were obtained using a T2∗-weighted multiband echo-planar imaging sequence (TR = 987 ms, voxel size of 2 × 2 × 2 mm, 72 slices). The total acquisition time was 7 minutes and 34 seconds. During the resting-state scan, participants were instructed to fixate on a cross on a screen and let their mind wander.

### Imaging Analyses

Results included in this article come from analyses performed using CONN (19) release 22.a (RRID:SCR_009550) (20) and SPM (21) (RRID:SCR_007037) release 12.7771.

### Preprocessing and Denoising

Preprocessing included realignment with correction of susceptibility-distortion interactions, outlier detection with a conservative framewise displacement above 0.5 mm or global blood oxygen level–dependent (BOLD) signal changes above 3 SDs, segmentation and direct Montreal Neurological Institute space normalization, and smoothing. The functional data were then denoised using a standard denoising pipeline (22) and subjected to quality control.

### Functional Connectivity MVPA

Two models were created, SP versus CG and pre- versus posttreatment.

On the first statistical level, fc-MVPAs (15) were performed to estimate eigenpatterns for the SP versus CG model and for the pre vs. post model characterizing the principal axes of heterogeneity in functional connectivity across participants. From these eigenpatterns, 15 or 7 associated eigenpattern-score images were derived for each individual participant characterizing their brainwide functional connectome state. Individual functional connectivity values were computed from the matrices of bivariate correlation coefficients between the BOLD time series from each pair of voxels, estimated using a singular value decomposition of the *z* score–normalized BOLD signal with 64 components separately for each participant (15).

Group-level analyses were performed using a general linear model (GLM) (22). For each individual voxel, a separate GLM was estimated, with first-level connectivity measures at this voxel as dependent variables. Handedness, sex, and age were used as control variables in the SP versus CG model, and handedness and sex were used as control variables in the pre vs. post model. Voxel-level hypotheses (SP vs. CG and pre vs. post) were evaluated using multivariate parametric statistics. Inferences were performed at the level of individual clusters and based on nonparametric statistics using threshold-free cluster enhancement (TFCE) with default values for *F* tests (*H* = 1, *E* = 0.5, and *H*_min._ = 1) (22,23) and 1000 residual-randomization iterations. A TFCE-score threshold of familywise error–corrected *p* (*p*_FWE_) < .05 was applied.

### Illustration of Differences in Connectivity Patterns (Network View)

Differences in connectivity patterns between the fc-MVPA result clusters and the rest of the brain were presented as effect size maps, using all voxels in the selected clusters as seeds.

### Post Hoc Analyses

In post hoc analyses, the clusters identified in the fc-MVPA were used as seed regions to determine patterns of connectivity. Seed-based connectivity (SBC) analyses were then used to test which parts of the effect size maps survived standard parametric inferential statistical tests with the following thresholds: voxel-level *p* > .001, cluster-level *p*_FWE_ < .05. The resulting clusters were stored as binary ROIs, and the mean connectivity values (mean difference from 0 per group [SP, CG] or condition [pre, post]) were extracted for visualization and for post hoc correlational analyses with clinical data.

### ROI-to-ROI Analyses

The binary ROIs formed in one model from the result clusters of the fc-MVPA and SBC analyses were used in the other model to perform ROI-to-ROI connectivity (RRC) analyses. In this way, it was tested whether the connectivity strengths (the average deviation of connectivity from 0) between the ROIs identified in the SP versus CG model also showed differences in the pre vs. post model and vice versa.

### Correlations Between Connectivity Values and Clinical Data

Spearman correlations were used to test for associations between the extracted mean connectivity values and performance on the Behavioral Avoidance Test (BAT) (24) and the Fear of Spiders Questionnaire (FSQ) (25). The BAT assesses real-life avoidance behavior. Participants were instructed to enter a room with a spider in a transparent box, walk up to the box, and allow the spider to crawl on their hand for 20 seconds. Participants could stop at any time. The remaining time and the distance to the box were recorded. Higher scores on a scale between 1 and 12 indicate how successfully the task was completed. The FSQ is a self-report questionnaire that assesses fear of spiders. Higher scores indicate greater fear, with a possible range of 0 to 108.

### Network Characterization

Finally, all clusters were tested for overlap with a set of 7 canonical brain networks (26).

## Results

The main results are summarized below. Detailed additional information can be found in the Supplement.

### Quality Control

Quality control showed high data quality for all analyses (Figures S1–S6).

### fc-MVPA Group-Level Model (SP vs. CG)

**Figure 1:**
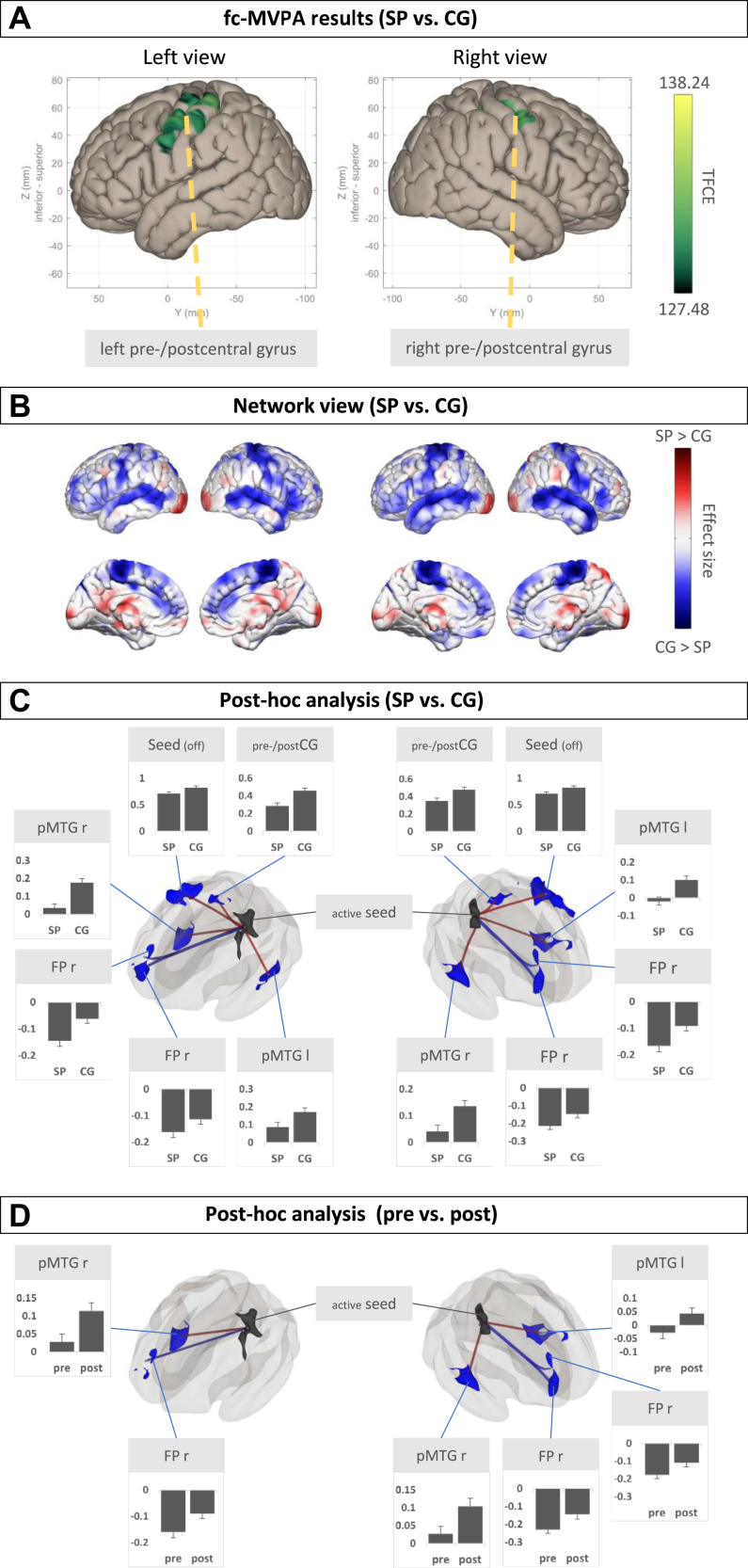
Group-level contrast specific phobia (SP) vs. control group (CG). **(A)** Results from the functional connectivity multivariate pattern analysis (fc-MVPA) on a left and right view of a Montreal Neurological Institute standard brain, **(B)** network view, effect size maps (SP vs. CG), and **(C)** post hoc seed-based connectivity analyses with fc-MVPA clusters as seed regions. All clusters were saved as regions of interest (ROIs) and further used in ROI-to-ROI analysis to extract connectivity values. **(D)** ROIs from the SP > CG contrast tested for differences in the pre > post contrast. ROI color: Black indicates the (active) seed region, and blue indicates CG > SP **(C)** and post > pre **(D)**. Connection color: Red lines indicate positive connections (positive correlations), and blue lines indicate negative connections (anticorrelations). Bar graphs show the connectivity values (in this case, the mean difference from 0) per group (SP vs. CG) **(C)** or condition (pre vs. post) **(D)**. Error bars indicate SEs. FP, frontal pole; l, left; pMTG, posterior division of the middle temporal gyrus; pre-/postCG, precentral gyrus, postcentral gyrus; r, right; TFCE, threshold-free cluster enhancement.

The fc-MVPA revealed 2 clusters, shown in the order of cluster size. Cluster 1 (742 voxels) is located in the left hemisphere, and cluster 2 (550 voxels) is located in the right hemisphere. Both clusters cover parts of the precentral and postcentral gyri (Figure 1A).

### Network View of Effect Sizes

The effect size maps of functional connectivity of the fc-MVPA clusters showed higher connectivity in the CG compared with the SP group mainly in pre- and postcentral regions, parts of the prefrontal, cingulate, and paracingulate cortices. Higher connectivity in the SP group compared with the CG was particularly evident in the visual regions but with considerably smaller effect sizes (Figure 1B).

### Post Hoc SBC Analysis

Using the left fc-MVPA result cluster as seed region, the analysis revealed 3 clusters with lower connectivity in the SP compared with the CG. Cluster 1 (491 voxels) mainly covers the right posterior division of the middle and superior temporal gyri (positive connectivity). Cluster 2 (208 voxels) covers parts of the left and right pre- and postcentral gyri (anticorrelation). Cluster 3 (201 voxels) covers parts of the right frontal pole (anticorrelation) (Figure 1C). No clusters were identified that showed higher connectivity in the SP group compared with the CG.

Using the right fc-MVPA cluster as seed region, the SBC analysis yielded 2 clusters with lower connectivity in the SP group compared with the CG. Cluster 1 (419 voxels) covers parts of the left posterior division of the middle and superior temporal gyri as well as temporo-occipital parts of the middle temporal gyrus (positive connectivity). Cluster 2 (318 voxels) covers parts of the right frontal pole (anticorrelation) (Figure 1C).

No clusters were identified that showed higher connectivity in the SP group than in the CG.

### RRC Post Hoc Analysis

The 5 clusters from both SBC analyses were saved as ROIs to visualize the direction of the effects in both models, SP versus CG and pre vs. post.

#### SP Versus CG Model

The SP group showed lower connectivity values between the seed regions and the ROIs than the CG. There was also positive connectivity between the seed regions, the temporal ROIs, and the pre- and postcentral gyrus ROI, as well as between the 2 fc-MVPA seed regions. In contrast, there were anticorrelations (negative connectivity values) between the seed regions and the frontal regions (Figure 1C).

#### Pre Versus Post Model

The RRC showed a significant difference between the seed regions and most of the target ROIs, which was smaller in the precondition than in the post condition (Figure 1D). Therefore, the connectivity values in the SP group after treatment were similar to the connectivity values in the CG at baseline (Figure 1C).

### Correlation Analyses, Cluster Overlap, and Sensitivity Analysis

Correlation analyses with clinical data (FSQ, BAT) showed no significant results.

**Figure 2:**
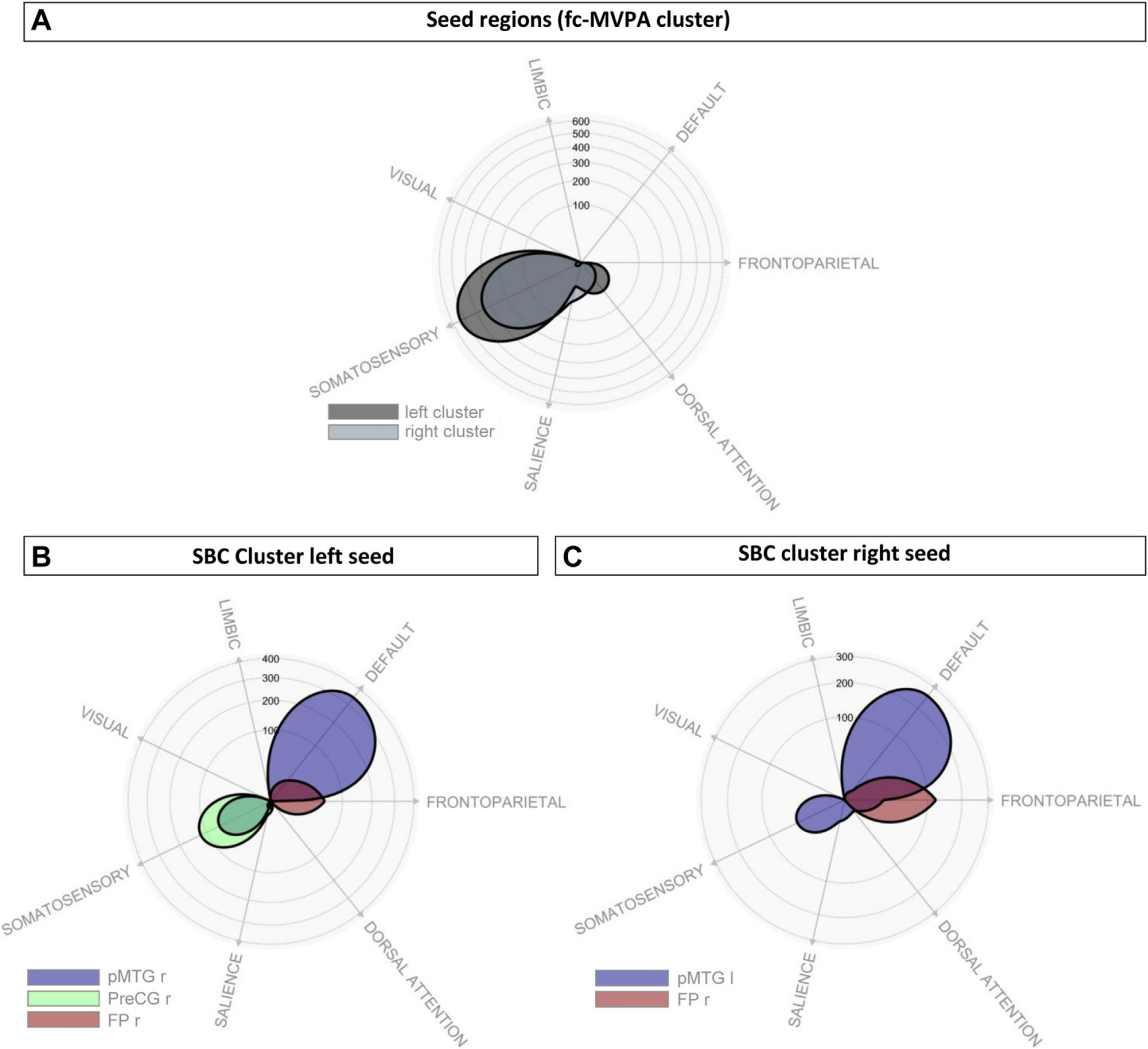
Polar display showing the overlap between each significant cluster of the specific phobia vs. control group model and a set of canonical brain networks. **(A)** Functional connectivity multivariate pattern analysis (fc-MVPA) clusters (seed regions), **(B)** negative correlated network clusters from the left seed region, and **(C)** anticorrelated network clusters from the right seed region. FP, frontal pole; l, left; pMTG, posterior division of the middle temporal gyrus; PreCG, precentral gyrus; r, right; SBC, seed-based connectivity.

Cluster overlap with canonical brain networks is presented in Figure 2 and Table S2.

See the Supplement for the results of an additional sensitivity analysis.

### fc-MVPA Pre Versus Post Treatment Model

**Figure 3:**
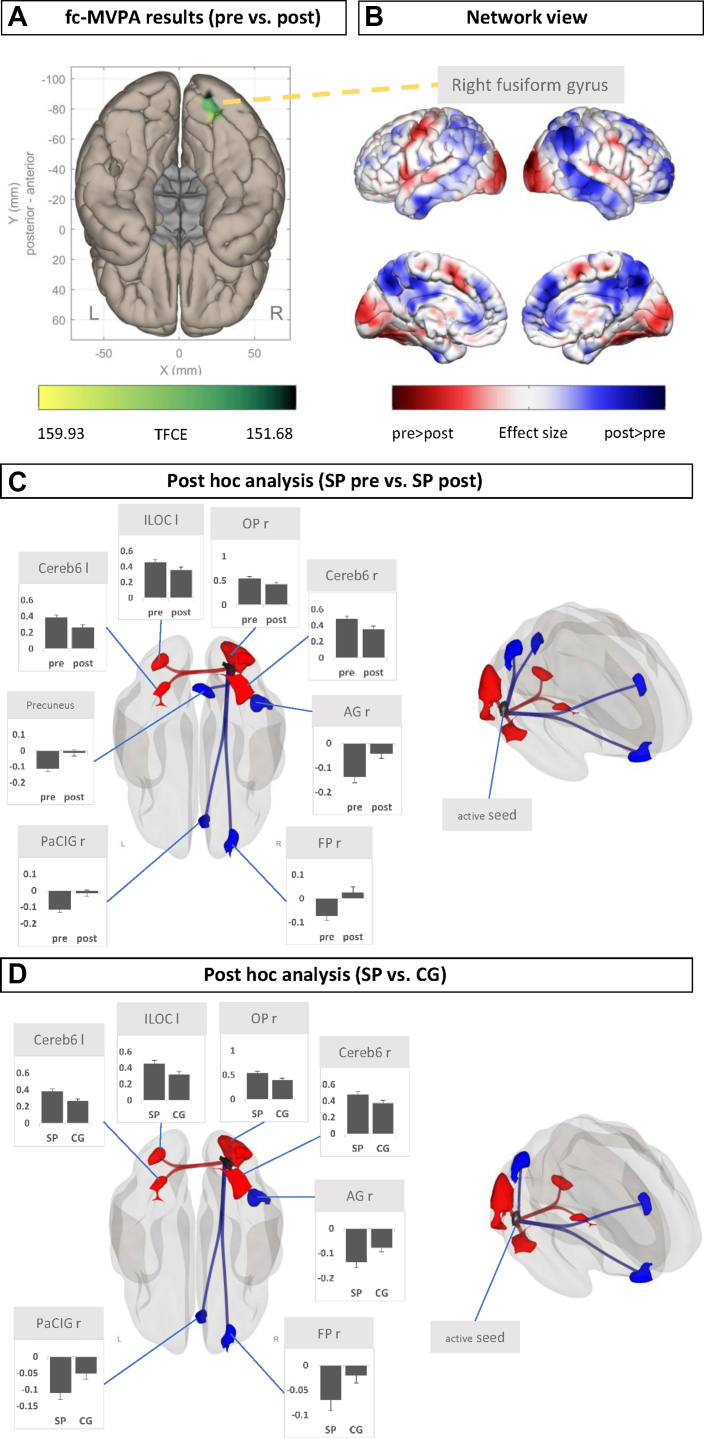
Condition-level contrast from the pre vs. post one-session exposure treatment comparison. **(A)** Results from the functional connectivity multivariate pattern analysis (fc-MVPA) on an inferior view of a Montreal Neurological Institute standard brain, **(B)** network view, effect size maps (SP vs. CG), and **(C)** post hoc seed-based connectivity analyses with fc-MVPA clusters as seed regions. All clusters were saved as regions of interest (ROIs) and further used in ROI-to-ROI analysis to extract connectivity values. **(D)** ROIs from the pre > post contrast tested for differences in the specific phobia (SP) > control group (CG) contrast. ROI color: Black indicates the (active) seed region, red indicates pre > post **(C)** and SP > CG **(D)**, and blue indicates post > pre **(C)** and CG > SP **(D)**. Connection color: Red lines indicate positive connections (positive correlations), and blue lines indicate negative connections (anticorrelations). Bar graphs show the connectivity values (in this case, the mean difference from 0) per condition, pre vs. post **(C)**, or group, SP vs. CG **(D)**. Error bars indicate SEs. An additional figure showing the location of the cerebellar clusters can be found in the Supplement (Figure S4). AG, angular gyrus; Cereb6, cerebellum 6; FP, frontal pole; ILOC, inferior division of the lateral occipital gyrus; L/l, left; OP, occipital pole; PaCIG, paracingulate gyrus; R/r, right; TFCE, threshold-free cluster enhancement.

The fc-MVPA analysis revealed 1 cluster (149 voxels) that mainly covers the right occipital fusiform gyrus (Figure 3A).

### Network View of Effect Sizes

The effect size maps of functional connectivity of the fc-MVPA cluster as seed region showed higher connectivity of the pre- versus posttreatment condition mainly in occipital, occipitotemporal, anterior superior temporal, and precentral regions. A higher connectivity of the post- versus pretreatment condition was mainly seen in prefrontal, cingulate, and parietal regions. (Figure 3B).

### Post Hoc SBC Analysis

Using the fc-MVPA result cluster as seed region, the analysis revealed 4 clusters with positive connectivity and higher values in the pre- versus posttreatment condition, shown in the order of cluster size.

Cluster 1 (1150 voxels) is located in the right hemisphere and mainly covers the occipital pole and inferior lateral parts occipital cortex. Cluster 2 (317 voxels) mainly covers the right cerebellum 6 and the right temporal occipital fusiform cortex. Cluster 3 (208 voxels) covers parts of the left inferior and superior lateral occipital gyri. Cluster 4 (155 voxels) covers parts of the left cerebellum 6 and the left occipital and temporal occipital gyri.

In addition, 4 anticorrelated clusters with lower connectivity in the pre vs. post condition were identified. Cluster 1 (328 voxels) mainly covers parts of the right angular gyrus, superior parts of the right lateral occipital cortex, and the superior parietal lobule. Cluster 2 (283 voxels) covers parts of the right frontal pole. Cluster 3 (250 voxels) covers parts of the right precuneus cortex. Cluster 4 (206 voxel) covers parts of the right paracingulate gyrus and a small proportion of the right superior frontal gyrus (Figure 3C).

### RRC Post Hoc Analysis

The 8 clusters from the SBC analyses were saved as ROIs to visualize the direction of the effects in both models, pre vs. post and SP versus CG.

#### Pre- Versus Posttreatment Model

Higher connectivity values in the pre- vs. posttreatment condition were observed in the positively connected ROIs of the occipital and cerebellar regions. In contrast, lower connectivity values in the pre vs. post treatment condition were observed in the anticorrelated frontal and parietal ROIs and the precuneus (Figure 3C).

#### SP Versus CG Model

The RRC analysis also showed a significant difference between the seed regions and the target ROIs, which was larger in the SP group than in the CG. With the exception of the precuneus, the connectivity values were lower in the SP compared with the CG in the anticorrelated ROIs (Figure 3D). This model showed that the CG connectivity values at baseline were largely comparable to those of the posttreatment condition in the SP group (Figure 3C).

### Correlation Analyses and Cluster Overlap

Correlation analyses with clinical data (FSQ, BAT) showed no significant results.

**Figure 4:**
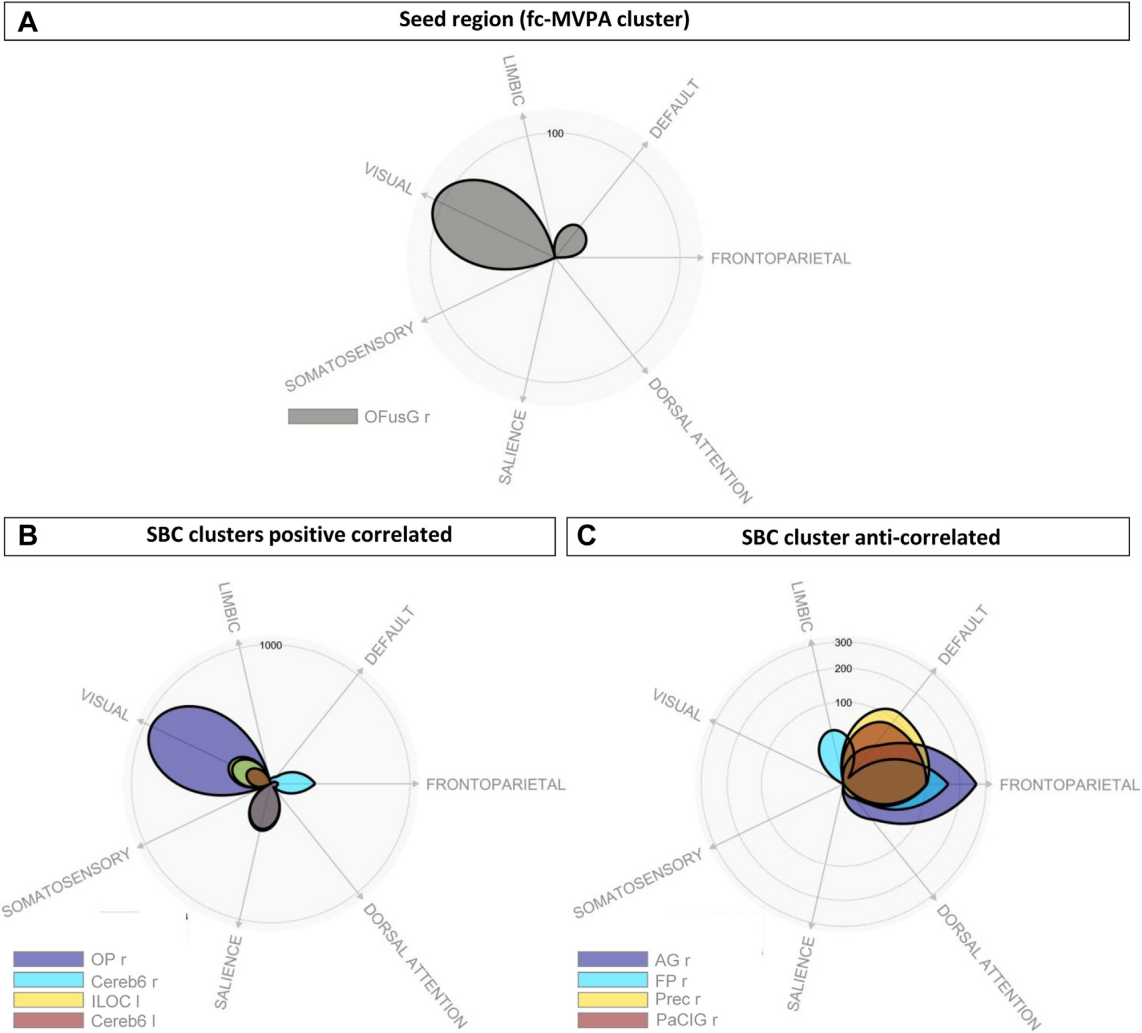
Polar display showing the overlap between each significant cluster of the pre vs. post model and a set of canonical brain networks. **(A)** Functional connectivity multivariate pattern analysis (fc-MVPA) cluster (seed region), **(B)** positive correlated network clusters, and **(C)** anticorrelated network clusters. AG, angular gyrus; Cereb6, cerebellum 6; FP, frontal pole; ILOC, inferior division of the lateral occipital gyrus; l, left; OFusG, occipital fusiform gyrus; OP, occipital pole; PaCIG, paracingulate gyrus; Prec, precuneus; r, right; SBC, seed-based connectivity.

Overlap with canonical networks is presented in Figure 4 and Table S3.

## Discussion

Using fc-MVPA, we were able to identify clusters in the pre- and postcentral gyri that showed different connectivity patterns between the SP group and CG. After a one-session exposure treatment, the connectivity patterns of the SP group were similar to those of the CG at baseline.

The group comparison showed a weaker connection between sensorimotor clusters and the temporal subsystem of the default network in the SP group compared with the CG. These regions have not been the focus of SP research, possibly because they are not activated by common stimuli such as affective pictures. However, a recent study using resting-state ICA found similar results as a function of trait anxiety in an adolescent sample (27). As input and output regions (perceiving and acting), the sensorimotor cortices react to rapid changes in the environment, whereas the regions of the default network enable cognitive processing independent of the here and now. A connection between these sensorimotor and temporal regions has been reported previously (28). The default network is thought to form context-dependent models of situations by receiving information from the environment via the sensory regions and comparing it with previous experience and intrinsic expectations (29). Temporal nodes are related to memory retrieval and abstract thinking about situations (30). In this way, expectations can be compared with the actual situation of the external world, or the output (acting) regions can be prepared, e.g., for expected changes in the external world. Information from systems at different hierarchical levels is brought together via transmodal integration nodes, such as those in the temporal subsystem (28). Our data show lower coupling between the regions of perception and action (sensorimotor regions) and the integration nodes in the SP group but an increase at posttreatment measurement. A poorer exchange of information between these regions could lead to an incorrect comparison of the situation with previous experiences in individuals with SP and make it more difficult to predict the environment correctly. Stronger connections in the posttreatment measurement may be associated with better comparison of the environment with expectations and better prediction of situations. The analysis also showed an anticorrelation between the sensorimotor regions and the right frontal pole. The anticorrelation was lower in the SP group than in the CG. In the posttreatment measurement, the anticorrelation was reduced and was comparable to the baseline measurement of the CG. Anticorrelations are difficult to interpret, especially in regions that have a large Euclidean (31) or geodesic distance (32) and communicate at different frequencies, such as the sensorimotor regions (fast transient signals) and higher-order regions such as the fontal pole (slow sustained responses) (33). For example, graph models showed a positive correlation between the shortest path length and the strength of functional connectivity anticorrelation, in that longer paths were associated with higher anticorrelation, possibly due to phase delays between distant regions (31). Therefore, a reduction in anticorrelation with treatment could indicate increased network efficiency and shorter path lengths, reflecting better phase synchronization in large-scale communication. Furthermore, the nature of anticorrelations may be state dependent, indicating that a region’s connected counterparts may alternate within a single resting scan (34). Thus, in the CG and in the posttreatment measurement of the SP group, the frontal pole cluster might have more often been involved in controlling the sensing and acting processes of the sensorimotor clusters. Future studies should test these assumptions using graph models and dynamic connectivity measures. The frontal pole clusters identified in our study were strongly related to the frontoparietal network, and previous studies have linked the frontal pole to a number of higher-order functions such as action motor learning and emotional control (35,36). A reduction in anticorrelation in the SP group at postmeasurement may reflect a higher frequency of integration of frontal pole regions with sensorimotor regions, as well as a higher phase synchronization, which could improve the correct interpretation of environmental events as well as emotional and cognitive action control.

The fc-MVPA of the pre vs. post model identified 1 cluster within the right occipital fusiform gyrus. The post hoc analyses showed positive connections in numerous other regions of the visual system and parts of the cerebellum, with stronger connectivity in the SP group than in the CG. The connectivity values at postscan were lower than at prescan and were similar to the baseline values of the CG. Higher connectivity of visual areas in the SP group compared with the CG was also shown in another voxelwise study (37). Increased connectivity and activity is thought to be a sensitization of the visual system to phobic stimuli [e.g., (37, 38, 39)]. Therefore, the reduction in connectivity in the visual system of the SP group at postscan could indicate desensitization. The role of the cerebellum is largely unclear, but it may be involved in regulating anxious behavior (27,40).

In the pre vs. post model, we also found anticorrelations between the cluster in the visual system and higher-order regions such as the right frontal pole, right angular gyrus, and paracingulate gyrus, which could mainly be assigned to the frontoparietal and the default network. Following the reasoning in the previous section on anticorrelations, we hypothesize that the reduction in connectivity in the visual regions is related to improved communication with higher-order control regions. It should be emphasized that the anticorrelation between the visual regions and the right frontal pole actually shifts toward positive connectivity at postscan. Increased communication between these control regions and the visual system may counteract the misattribution of visual attention and allow more accurate perception and prediction of the visual environment. In fact, the right frontal pole cluster in this analysis matches the results of a meta-analysis linking this region to emotional action control (36).

Although the main analyses (SP vs. CG and pre vs. post) yielded different results, it should be noted that the reported results depend on the type and stringency of the correction for multiple testing. The uncorrected effect size maps (Figures 1B and 3B) and sensitivity analysis (Figure S10) show a more extensive pattern of altered connectivity, such as visual regions, in the SP versus CG model, explaining the significant results of the more sensitive exploratory RRC analysis (Figures 1D and 3D).

### Limitations

Without postmeasurement of the CG, we cannot exclude the possibility that the changes in connectivity (pre-post changes in SP) are due to unknown factors other than treatment. To draw causal conclusions, future studies should use randomized controlled designs.

Furthermore, the time interval between baseline, treatment, and postscan was several weeks, but recent studies have shown that the functional connectome and treatment effects are stable over months (41,42).

Finally, no correlations were found between connectivity metrics and clinical data. This is consistent with other studies (14,41) and suggests that clinical scores of symptom severity do not necessarily reflect brain function of basic cognitive processes.

### Conclusions

Our study provides evidence that there is an increase in functional connectivity between sensorimotor regions and the integration nodes of the default network, while there is a concurrent reduction in connectivity within the visual system from pre- to posttreatment measurements. Furthermore, the visual and sensorimotor systems show reduced anticorrelations with different areas of the frontoparietal network. These changes in functional connectivity between sensory and motor areas with higher-order regions may improve cognitive-affective control of sensory and motor areas, leading to better perception and environmental prediction of mentally constructed scenes and thus improved action control. The posttreatment measurements were comparable to those of the CG at baseline. However, given the lack of posttreatment data in the CG, it remains uncertain whether our results can specifically be attributed to treatment.

## Supplementary Material

Supplemental Methods, Results, Discussion, Figures S1–S10, and Tables S1–S4

Key Resources Table
