# Supplementary material for: Brainwide Analysis of Functional Connectivity Patterns in Specific Phobia and Its Treatment
Source: Biol Psychiatry Glob Open Sci. 2025 Jul 8;5(6):100562. doi: 10.1016/j.bpsgos.2025.100562 (PMC12390938; doi:10.1016/j.bpsgos.2025.100562)
Supplement: Supplemental Methods, Results, Discussion, Figures S1–S10, and Tables S1–S4 [file mmc1.pdf]

SUPPLEMENTARY INFORMATION

Brainwide Analysis of Functional Connectivity Pattern in Specific Phobia and Its Treatment

Muehlhan *et al.*

Content

Supplementary methods 3
Procedure 3
Study sample 3
Diagnostic Assessments 4
Fear of Spiders Questionnaire 4
Behavioral Avoidance Test (BAT) 4
Treatment 4
Image acquisition and analyses 5
Image acquisition 5
fMRI analyses 6
Preprocessing 6
Denoising 6
Functional connectivity MultiVariate Pattern Analysis (fc-MVPA) 7
Seed-based connectivity (SBC) analyses 7
ROI-to-ROI (RRC) analyses 8
Quality Control 9
Quality Control: Group-Analysis (SP vs. CG) 9
Quality Control: Within-analysis (pre vs. post) 11
Supplementary Results 13
Detailed description of result clusters 13
'SP vs. CG' model 13
Post-hoc seed based connectivity (SBC) analysis 13
Overlap with canonical Large-Scale Brain Networks 14
'Pre vs. post' treatment model 14
Post-hoc seed based connectivity (SBC) analysis 14
Visualization of the result clusters including the subcortical and cerebellar regions 15
Overlap with canonical Networks within analysis (pre-post) 16
Requested additional analyses 16
Methods, Results and Discussion 16
Treatment response rate and connectivity strength 16
Sensitivity analysis: excluding SP participants with comorbidity 16
Sensitivity analysis: including SP participants without post-measurement 20
Supplementary Discussion 25
References 26

Supplementary methods

Procedure

Participants were invited to the baseline assessment (T1), which was conducted on two separate days for logistical reasons and to reduce the burden on participants. The first assessment day (T1a) consisted of a virtual decision-making game and a battery of tasks testing various cognitive functions (both tasks are not included here). In addition, questionnaires on clinical characteristics, cognitive control and volitional behavior were administered. In addition, questionnaires and interviews on clinical characteristics, cognitive control and volitional behavior were administered. The Behavioral Avoidance Test (BAT) was also administered (see below). The second examination day (T1b) took place one week later and began with a 45-minute habit induction task outside the MRI scanner. The MRI measurements began with a 20-minute habit strength measurement (not included here). After the task, two resting state scans were performed (the first with a standard EPI sequence (TR 3000 ms) for comparability with another project not considered here, and a fast multiband resting state EPI sequence (see below)). An anatomical sequence was recorded after the resting state measurements. All investigations were conducted at the Neuroimaging Centre of the Faculty of Psychology, TUD Dresden University of Technology.

At a further appointment (T2) approximately five weeks (mean: 5.74; SD: 4,26) after the fMRI scan, participants in the SP group received a one-session exposure treatment (see below).

The SP group was then assessed again (T3) approximately 15 weeks (mean: 15.13; SD: 7.83) after the baseline measurement, on two days identical to the baseline examination (T3a, T3b).

Study sample

Inclusion criteria were age between 18 and 50 years and manifest DSM-5 defined spider phobia with at least mild suffering or impairment (phobia group) or no lifetime psychopathology (comparison group), as assessed by standardized clinical interview (DSM-5 adapted research version (16)) of the (DIA-X/M-CIDI; (17)).

Exclusion criteria were: psychopharmacological medication or psychotherapeutic intervention in the previous four weeks, heavy nicotine use (> 10 cigarettes per day or equivalent), severe comorbidity (major depression or mania, psychosis) or suicidality, and MRI-related exclusion (e.g. non-MRI-compatible implants). In addition, phobic subjects were excluded if another specific phobia subtype of greater severity was present at the time of inclusion or if exposure therapy was not indicated (e.g. due to severe heart disease). Subjects were fully informed about the procedures of the study, and written informed consent was obtained.

The total study sample consists of GC: N = 82, SP: N = 110. While the data were complete for the CG group, there were numerous drop-outs and missing data in the SP sample:

- Dropout before fMRI baseline measurement: N = 10
- Dropout before fMRI post treatment measurement: N = 14
- Resting-state multi-band sequence data not complete or not usable: N = 14

The SP group considered in the main analyses 'SP vs. CG' and 'pre vs. post' includes only participants who completed treatment (N = 72), as this simplifies the description of the extensive methodology and facilitates the interpretation of the results.

On request, additional sensitivity analyses were conducted that includes 10 additional SP subjects for whom complete baseline (pre-scan) multiband resting state-data are available (N = 82) but no post-

scan data. Six of these participants did not complete treatment and a further four participants withdrew from the study before the second MRI scan.

Diagnostic Assessments

A clinical interview was used to assess the inclusion and exclusion criteria (DSM-5 adapted version of the Munich Composite International Diagnostic Interview (DIA-X/M-CIDI; (Wittchen & Pfister, 1997).

Fear of Spiders Questionnaire

The Fear of Spiders Questionnaire (FSQ; (Szymanski & O'Donohue, 1995)) was used to assess fear of spiders in general. The FSQ consists of 18 items such as 'I think a lot about spiders now' with a seven-point rating scale from [0] 'strongly disagree' to [6] 'strongly agree'. The total score was generated with a possible range of 0-108.

Behavioral Avoidance Test (BAT)

The BAT was used to assess real-life avoidance behavior (Olatunji & Deacon, 2008). A spider in a transparent container with a lid was placed on a table five meters from the entrance. Subjects were given written instructions explaining the test. They were instructed to enter the room, walk up to the container, remove the lid and allow the spider to crawl on their hand for 20 seconds if they wished. They were free to stop at any time if they did not wish to continue. Two pre- (15 min, 1 min before BAT) and three post-assessments (1 min, 15 min, 30 min after BAT) including an anxiety rating on a scale from 1 (not at all anxious) to 100 (totally anxious) with additional saliva samples to measure cortical response to the BAT were administered. To minimize the experimenter effect, the experimenters did not speak to the participants during the BAT and only signaled when to begin. During the approach, the experimenter remained in the background so as not to serve as a safety behavior. The task was stopped after 90 seconds by the experimenter or when the participants chose to stop the task. The remaining time and distance to the box were recorded, as well as a score using the following coding scheme:

0 – Refuses to enter the room
1 – Stops 5m before the container
2 – Stops 4m before the container
3 – Stops 3m before the container
4 – Stops 2m before the container
5 – Stops 1m before the container
6 – Stops right in front of the container
7 – Touches the container
8 – Removes the lid
9 – Puts one hand in the container
10 – Touches the spider
11 – Holds the spider, but for less than 20s / Tries to hold the spider, but for less than 20s*
12 – Holds the spider for 20s / Tries to hold the spider for 20s

Treatment

Individuals with specific phobias received single-session exposure-based therapy as proposed by Ost (Ost, 1989). This treatment intervention includes techniques from the full spectrum of cognitive behavioral therapy, but relies primarily on exposure and is limited to a single 3-hour session. Single-session treatment for specific phobias has been shown to be highly effective in numerous studies, with large effect sizes and response rates of around 75% (Choy et al., 2007; Wolitzky-Taylor et al., 2008; Zlomke & Davis, 2008).

The exact procedure in the current study was as follows: After the baseline assessment, the therapist explained the treatment rationale to the patient and obtained informed consent for the treatment. During the exposure session itself, the aim was to complete a series of treatment steps, the final step being for the patient to hold or touch the feared stimulus (here: spider) for one minute or longer. The therapist guided the patient by modelling the appropriate response. The therapy session ended after 3 hours or when the patient was able to complete the final step with only mild to moderate anxiety. Of course, the patient was allowed not to start the session or to end it early if he or she wished, after appropriate therapeutic encouragement to continue. The patient's ability to start and complete the session without dropping out, the time taken to complete the various steps, and the patient's ratings of subjective anxiety during these steps at the end of the session were used as outcome data in predicting treatment success. For the therapeutic approach itself, a reliable and standardized treatment manual was used (*Intensive one-session treatment of specific phobias*, 2012).

Table S1: Sample characteristics

	**SP (N = 72)**	**CG (N = 82)**	**statistic**
Age (SD)	25.5 (6.8)	23.8 (5.1)	$p = 0.076$
Female (%)	66 (91.7)	82 (90.2)	$\chi^2 = 0.759$
left handed (%)	6 (8.4)	3 (3.6)	$\chi^2 = 0.224$
FSQ score	78.93 (12.19)	2.46 (3.05)	$p < 0.001$
BAT score	6.10 (2.02)	11.41 (1.12)	$p < 0.001$
	**SP pre treatment**	**SP post treatment**	
FSQ	78.93 (12.19)	39.35 (20.09)	$p < 0.001$
BAT	6.10 (2.02)	8.58 (2.49)	$p < 0.001$

SP: Spider Phobia group; CG: control group; FSQ: Fear of Spiders Questionnaire; BAT: Behavioral Avoidance Test

Other DSM-5 diagnoses (past 12 months) in the SP group:

- Other Anxiety Disorder: N = 4
- Somatic Symptom or related Disorder: N = 2
- Obsessive Compulsive Disorder: N = 2
- Attention Deficit/Hyperactivity Disorder: N = 1
- Any Other Disorder: N = 9

Image acquisition and analyses

Image acquisition

Images were acquired using a 3-Tesla Trio-Tim syngo MR B17 whole-body scanner (Siemens, Erlangen, Germany) with a 32-channel head coil. Standard headphones with additional earplugs were used to reduce sound pressure. Structural imaging acquisition parameters were: (repetition time (TR): 2400 ms, echo time (TE): 2.19 ms, flip angle: α = 8°). Functional measurements were obtained using a T2* weighted gradient whole brain multi-band echo planar imaging (EPI) sequence. TR: 987 ms, TE: 32.6 ms, flip angle: α = 8°, Multi-band acceleration factor: 6. 450 whole brain volumes were acquired with a voxel size of 2x2x2 mm in 72 slices. The total acquisition time was 7 minutes and 34 seconds. During the resting state scan, participants were instructed to fixate a cross on a screen and let their thoughts wander.

fMRI analyses

The data were analyzed in two separate models. One for group analysis ‘SP vs. CG’ and one for the within design to analyze the effect of the one session exposure treatment ‘pre-post’.

In order to achieve maximum transparency and comparability with other studies, the following parts of the methods were written using the CONN-Methods function, which is subject to the Creative Common License CC0 1.0. Study specific information has been adapted or added as appropriate.

Results included in this manuscript come from analyses performed using CONN (Whitfield-Gabrieli & Nieto-Castanon, 2012) (RRID:SCR_009550) release 22.a (Nieto-Castanon & Whitfield-Gabrieli, 2022) and SPM (Penny et al., 2011) (RRID:SCR_007037) release 12.7771.

Preprocessing

Preprocessing: Functional and anatomical data were preprocessed using a flexible preprocessing pipeline (Nieto-Castanon, 2020) pp. 3-16 including realignment with correction of susceptibility distortion interactions, outlier detection, direct segmentation and MNI-space normalization, and smoothing. Functional data were realigned using SPM realign & unwarp procedure (Andersson et al., 2001) where all scans were coregistered to a reference image (first scan of the first session) using a least squares approach and a 6 parameter (rigid body) transformation (Friston et al., 1995), and resampled using b-spline interpolation to correct for motion and magnetic susceptibility interactions. Potential outlier scans were identified using ART (Whitfield-Gabrieli et al., 2011) as acquisitions with framewise displacement above 0.5 mm or global BOLD signal changes above 3 standard deviations (Power et al., 2014), and a reference BOLD image was computed for each subject by averaging all scans excluding outliers. Functional and anatomical data were normalized into standard MNI space, segmented into grey matter, white matter, and CSF tissue classes, and resampled to 2 mm isotropic voxels following a direct normalization procedure (Calhoun et al., 2017) using SPM unified segmentation and normalization algorithm (Ashburner, 2007; Ashburner & Friston, 2005) with the default IXI-549 tissue probability map template. Last, functional data were smoothed using spatial convolution with a Gaussian kernel of 8 mm full width half maximum (FWHM).

Denoising

Functional data were denoised using a standard denoising pipeline (Nieto-Castanon, 2020) pp. 17-25 including the regression of potential confounding effects characterized by white matter timeseries (10 CompCor noise components), CSF timeseries (5 CompCor noise components), motion parameters and their first order derivatives (12 factors) (Friston et al., 1996), outlier scans (below 120 factors for ‘SP vs. CG’ model; below 53 factors for the ‘pre vs. post’ model) (Power et al., 2014), session effects and their first order derivatives (2 factors), and linear trends (2 factors) within each functional run, followed by bandpass frequency filtering of the BOLD timeseries (Hallquist et al., 2013) between 0.008 Hz and 0.09 Hz. CompCor (Behzadi et al., 2007; Chai et al., 2012) noise components within white matter and CSF were estimated by computing the average BOLD signal as well as the largest principal components orthogonal to the BOLD average, motion parameters, and outlier scans within each subject's eroded segmentation masks. From the number of noise terms included in this denoising strategy, the effective degrees of freedom of the BOLD signal after denoising (Nieto-Castanon, 2022b) were estimated to range from 48.4 to 67.8 (average 66.3) across all subjects in the ‘SP vs. CG’ model and from 122.9 to 136.3 (average 133.3) across all subjects in the ‘pre vs. post’ model.

Functional connectivity MultiVariate Pattern Analysis (fc-MVPA)

Two models were created 'SP vs. HC' and 'pre- vs. post treatment'

On the first statistical level, fc-MVPA (Nieto-Castanon, 2022a) were performed. Therefore, the number of eigenpatterns to be examined must be determined. We decided to start using an approximate 10:1 ratio between the number of participants (N) in the analysis and the number of eigenpattern (k), in order to maintain a reasonable sensitivity to identify medium effects in a relatively large sample (15). For the 'SP vs. HC' model this results in (N=154; k=15), and for the pre-post analysis (N=72; k=7). The first 15 'SP vs. CG' model or 7 'pre vs. post' model eigenpatterns were estimated to characterize the principal axes of heterogeneity of functional connectivity between subjects. From these eigenpatterns, 15 or 14 associated eigenpattern-score images were derived for each individual subject characterizing their brain-wide functional connectome state. Eigenpatterns and eigenpattern-scores were computed separately for each individual seed voxel as the left- and right- singular vectors, respectively, from a singular value decomposition (group-level SVD) of the matrix of functional connectivity values between this seed voxel and the rest of the brain (a matrix with one row per target voxel, and one column per subject). Individual functional connectivity values were computed from the matrices of bivariate correlation coefficients between the BOLD timeseries from each pair of voxels, estimated using a singular value decomposition of the z-score normalized BOLD signal (subject-level SVD) with 64 components separately for each subject (Nieto-Castanon & Whitfield-Gabrieli, 2022).

Group-level analyses were performed using a General Linear Model (GLM (Nieto-Castanon, 2020b) pp. 63-82)). For each individual voxel a separate GLM was estimated, with first-level connectivity measures at this voxel as dependent variables. Handedness, sex and age were used as control variables in the 'SP vs. CG' model, and handedness and sex in the 'pre- vs. post' model. The adjustment for these covariates was necessary due to the imbalanced distribution of sex and handedness within the groups, as well as the substantial age range, which has been demonstrated to exert a significant effect on functional connectivity (Nieto-Castanon, 2022a; Tejavibulya et al., 2022; Zhang et al., 2016). Voxel-level hypotheses ('SP vs. CG' and 'pre vs. post') were evaluated using multivariate parametric statistics with random-effects across subjects and sample covariance estimation across multiple measurements. Inferences were performed at the level of individual clusters. Cluster-level inferences were based on nonparametric statistics using Threshold Free Cluster Enhancement (TFCE) with default values for F -Tests H=1, E=0.5, and Hmin=1 (Nieto-Castanon, 2020; Smith & Nichols, 2009), and 1000 residual-randomization iterations. A family-wise corrected p-FWE < 0.05 TFCE-score threshold was applied.

As the second level analysis for the 'pre vs. post' model did not yield significant results, we repeated the whole procedure but increased the sensitivity by extending the number of eigenvalues to 14, which corresponds to an N=72:k =14 ratio of 5:1 (Nieto-Castanon, 2022a).

On request, we performed a sensitivity analysis with 10 additional SP subjects who dropped out of the study after the baseline measurement.

Seed-based connectivity (SBC) analyses

First-level analysis SBC: Seed-based connectivity maps (SBC) were estimated characterizing the patterns of functional connectivity with two seed ROIs in the 'SP vs. CG' model and one seed ROI in the 'pre vs. post' treatment model. Functional connectivity strength was represented by Fisher-transformed bivariate correlation coefficients from a weighted general linear model (weighted-GLM (Nieto-Castanon, 2020) pp. 26-62), defined separately for each pair of seed and target voxels,

modeling the association between their BOLD signal timeseries. In order to compensate for possible transient magnetization effects at the beginning of each run, individual scans were weighted by a step function convolved with an SPM canonical hemodynamic response function and rectified.

Group-level analyses were performed using a General Linear Model (GLM (Nieto-Castanon, 2020) pp. 63-82). For each individual voxel a separate GLM was estimated, with first-level connectivity measures at this voxel as dependent variables (one independent sample per participant and one measurement per task or experimental group or condition. Handedness, sex and age were used as independent control variables in the 'SP vs. CG' model, and handedness and sex in the 'pre- vs. post' model. Voxel-level hypotheses were evaluated using multivariate parametric statistics with random-effects across subjects and sample covariance estimation across multiple measurements. Inferences were performed at the level of individual clusters (groups of contiguous voxels). Cluster-level inferences were based on parametric statistics from Gaussian Random Field theory (Nieto-Castanon, 2020, pp. 83-104; Worsley et al., 1996). Results were thresholded using a combination of a cluster-forming $p < 0.001$ voxel-level threshold, and a familywise corrected p-FWE < 0.05 cluster-size threshold.

ROI-to-ROI (RRC) analyses

First-level analysis ROI-to-ROI connectivity matrices (RRC) were estimated characterizing the patterns of functional connectivity with 9 ROIs (1 Seeds, 8 target ROIS) within the 'SP vs. CG' model and 7 ROIs (2 Seeds, 5 target ROIs) within the 'pre vs. post' model. Functional connectivity strength was represented by Fisher-transformed bivariate correlation coefficients from a weighted general linear model (weighted-GLM (Nieto-Castanon, 2020)pp. 26-62), defined separately for each pair of seed and target ROIs, modeling the association between their BOLD signal timeseries. In order to compensate for possible transient magnetization effects at the beginning of each run, individual scans were weighted by a step function convolved with an SPM canonical hemodynamic response function and rectified.

Group-level analyses were performed using a General Linear Model (GLM, (Nieto-Castanon, 2020), pp. 63-82)). For each individual voxel a separate GLM was estimated, with first-level connectivity measures at this voxel as dependent variables (one independent sample per subject and one measurement per task or experimental condition, if applicable), and groups or other subject-level identifiers as independent variables. Voxel-level hypotheses were evaluated using multivariate parametric statistics with random-effects across subjects and sample covariance estimation across multiple measurements. Inferences were performed at the level of individual clusters (groups of contiguous voxels). Cluster-level inferences were based on parametric statistics from Gaussian Random Field theory (Nieto-Castanon, 2020; Worsley et al., 1996). Results were thresholded using a combination of a cluster-forming $p < 0.001$ voxel-level threshold, and a familywise corrected p-FDR < 0.05 cluster-size threshold (Chumbley et al., 2010).

Finally, all clusters were tested for overlap with a set of seven canonical brain networks (Yeo et al., 2011). This function was available in CONNs newest release CONN v.22.v2407.

Quality Control

Quality Control: Group-Analysis (SP vs. CG)

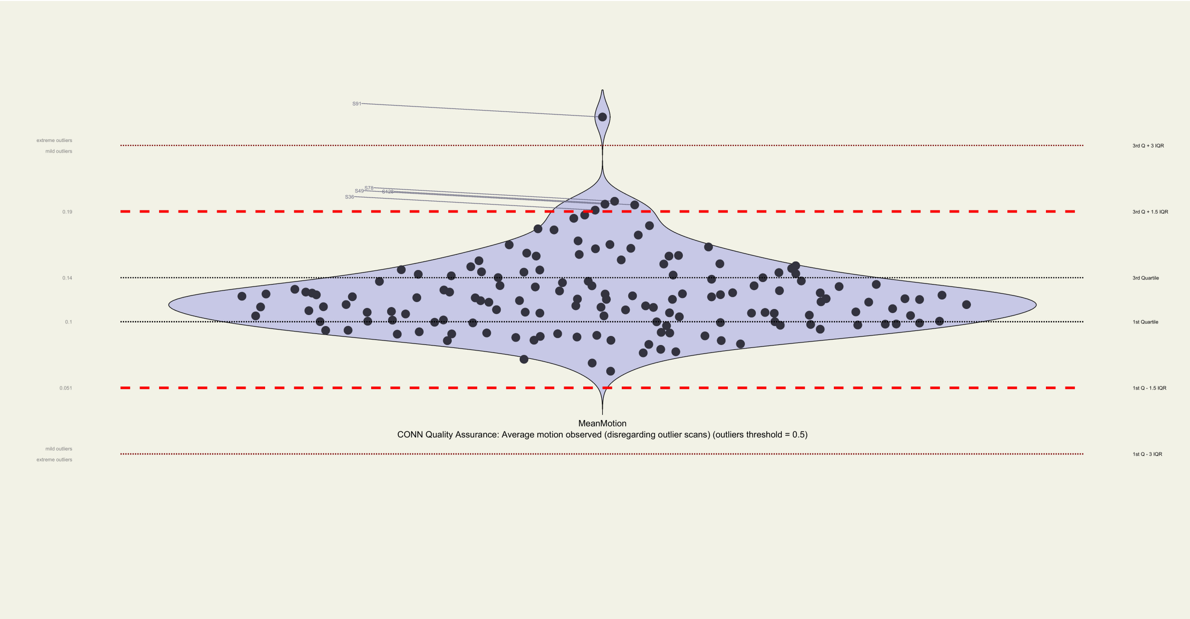

Figure S1: Violin Plots of the movement parameters (mean movements) of all 154 participants. The diagram shows that one participant (from the control group) is an extreme outlier.

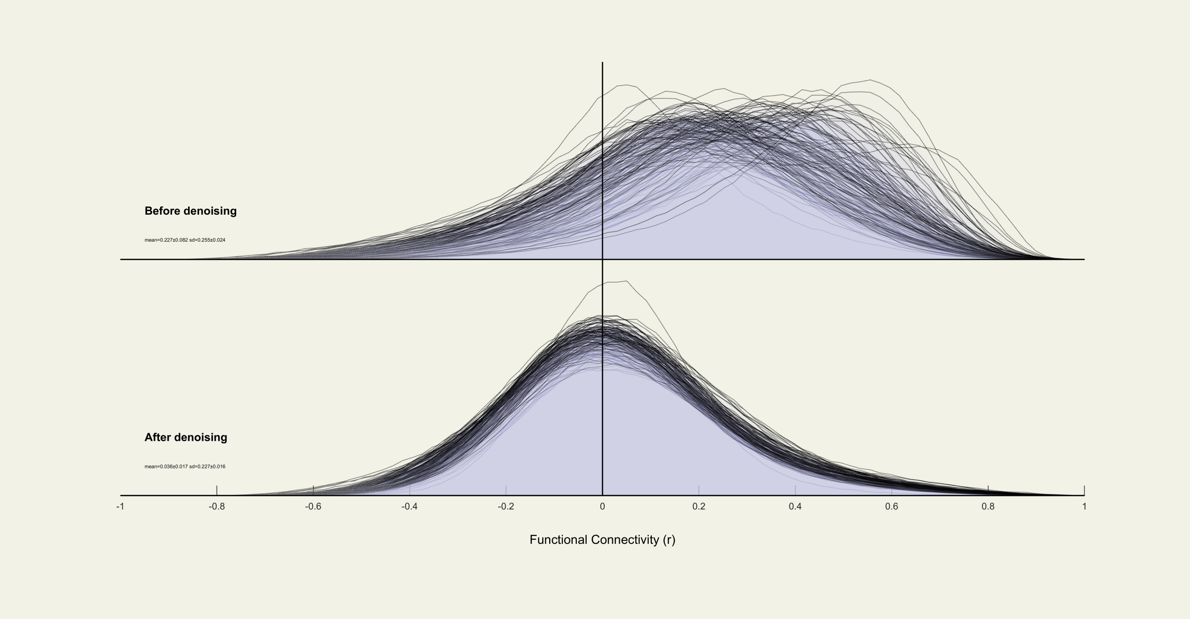

Figure S2: Functional Connectivity (FC) histograms of the entire sample. Distribution of functional connectivity (FC) values (correlation coefficients between 1000 voxel pairs within the grey matter) before (top) and after (bottom) denoising. The distributions appear well centered, indicating adequate denoising of the BOLD signal.

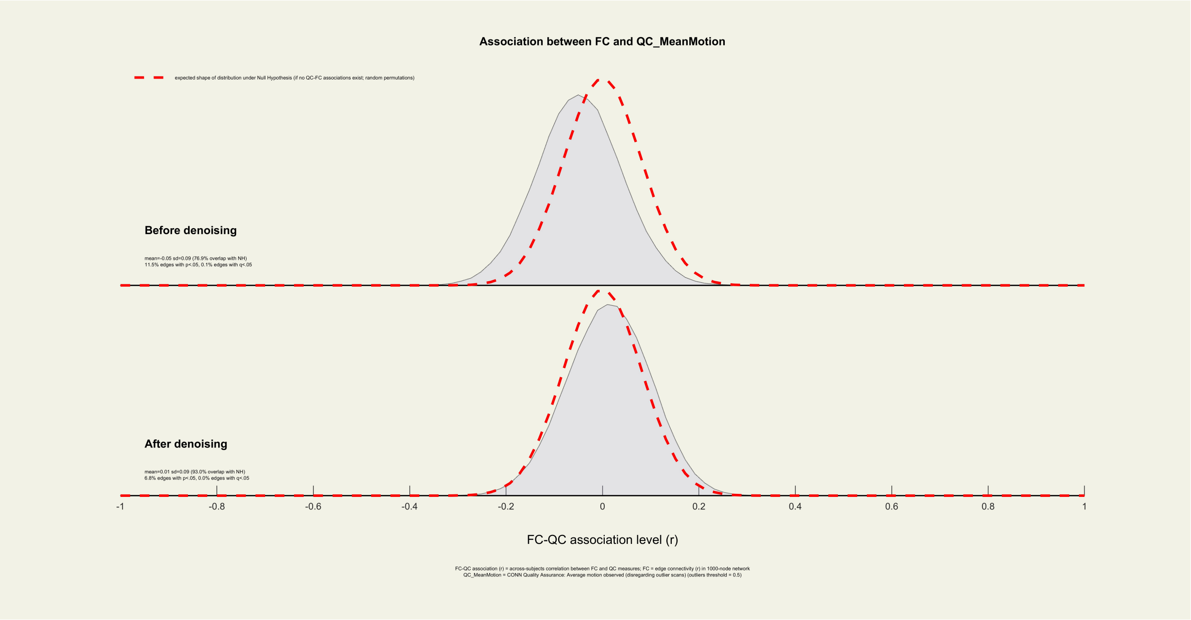

Figure S3: Distribution of Quality Control (QC) measures – Functional Connectivity (FC) values correlations. Distributions of Pearson correlation values between functional connectivity scores and a measure of movement (mean frame-related displacement), for all participants, before denoising (top) and after denoising (bottom). The red dashed lines show the expected distribution when there is no correlation between connectivity values and movement. The QC-FC distributions show a 93.0 % match with the expected distribution under the null hypothesis after denoising. The match rate is 95.9% after removing the outlier scans. A percentage match of over 95 % indicates an appropriate level of denoising of the BOLD signal.

Additional statistical analysis of QC measurements:

Mean motion standard deviation (SD) after denoising did not differ between groups (SP mean: 0.2241; CG mean: 0.2235) p=0.880.

Global Connectivity Correlation (GCOR) did not differ between groups (SP mean: 0.0223; CG mean: 0.0239) p=0.773

Number of valid scans after outlier removal from a total of 450 scans did not differ between groups: SP mean: 440.92; CG mean: 439.99) p = 0.654.

Quality Control: Within-analysis (pre vs. post)

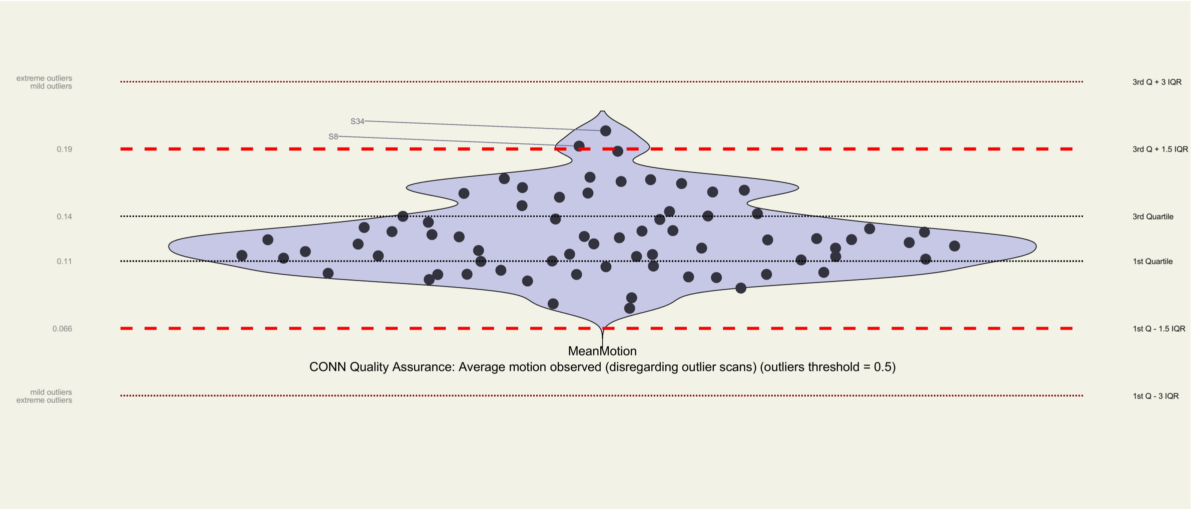

Figure S4: Violin Plots of the movement parameters (mean movements) of all 72 participants. The diagram shows no extreme outliers.

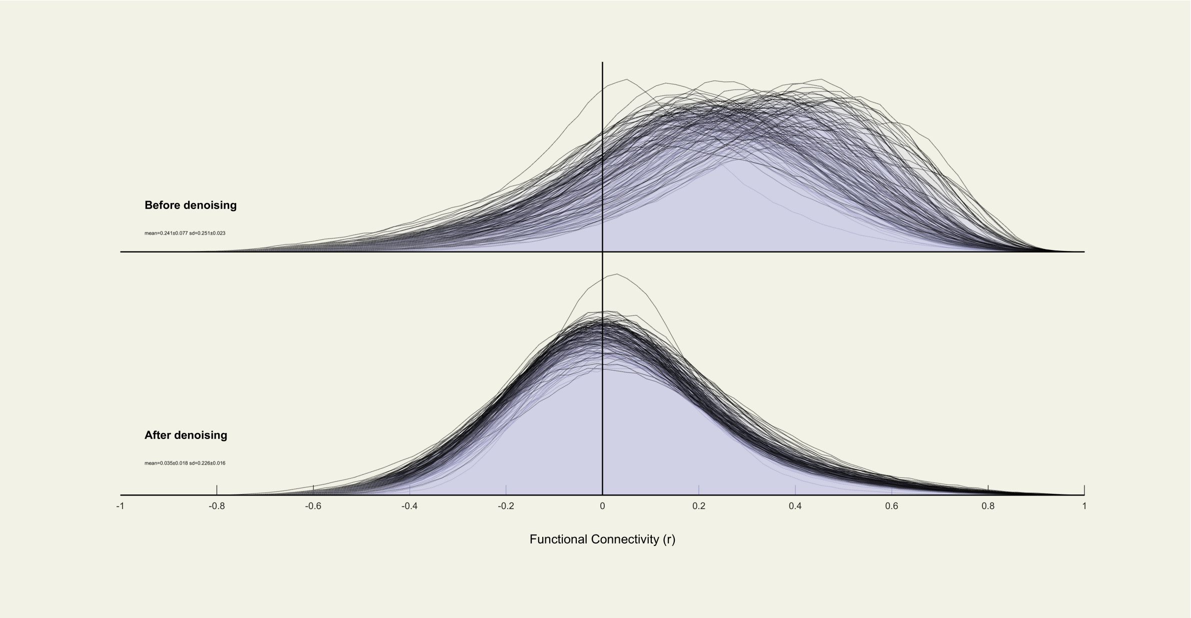

Figure S5: Functional Connectivity (FC) histograms of the pre- and post treatment measurements. Distribution of functional connectivity (FC) values (correlation coefficients between 1000 voxel pairs within the grey matter) before (top) and after (bottom) denoising. The distributions appear well cantered, indicating adequate denoising of the BOLD signal.

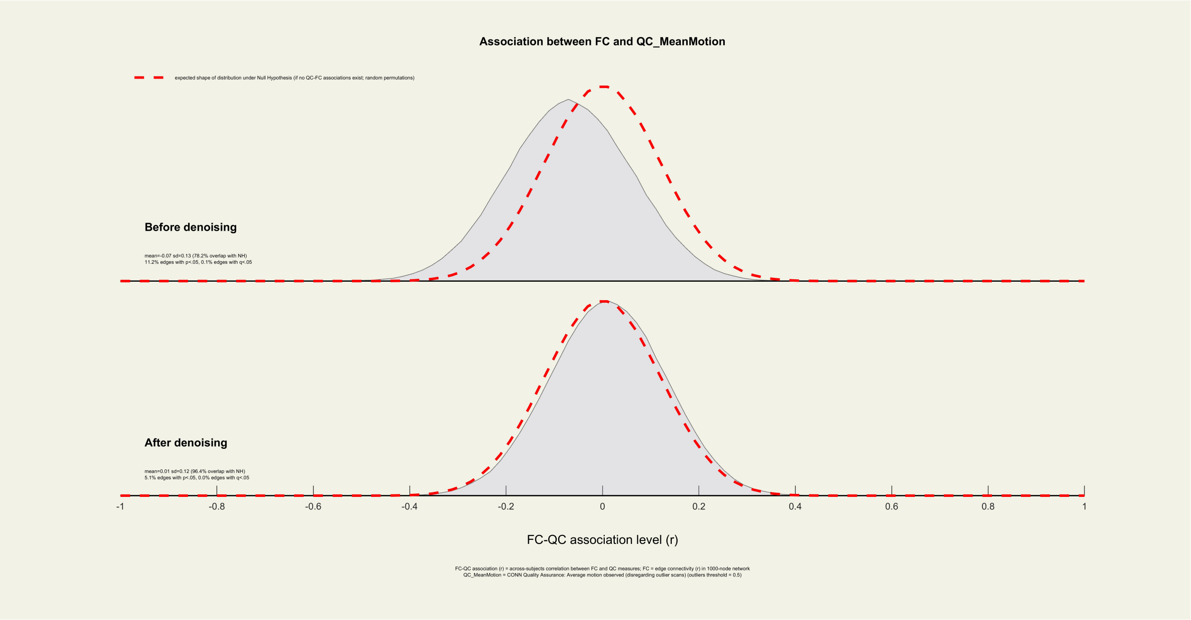

Figure S6: Distribution of Quality Control (QC) measures – Functional Connectivity (FC) values correlations of the pre- and post treatment measurements. Distributions of Pearson correlation values between functional connectivity scores and a measure of movement (mean frame-related displacement), for all participants, before denoising (top) and after denoising (bottom). The red dashed lines show the expected distribution when there is no correlation between connectivity values and movement. The QC-FC distributions show a 96.4 % match with the expected distribution under the null hypothesis after denoising. The match rate is 96.9% after removing the outlier scans. A percentage match of over 95 % indicates an appropriate level of denoising of the BOLD signal.

Additional statistical analysis of QC measurements:

Mean motion standard deviation (SD) after denoising did not differ between pre and post scans (pre mean: 0.2265; post mean: 0.2250) p=0.729

Global Connectivity Correlation (GCOR) did not differ between pre and post scans (pre mean: 0.0227; post mean: 0.0218) p=0.588

Number of valid scans after outlier removal from a total of 900 scans (450 before and 450 after treatment): SP mean: 881.25. The number of outliers is determined during scrubbing in the pre-processing stage, where the pre- and post-scans are processed together. Therefore, it is not possible to separate pre- and post-valid scans.

Supplementary Results

Detailed description of result clusters

‘SP vs. CG’ model

Fc-MVPA results

<u>Left Cluster: x: -36 y: -26 z: +50; size 742 voxel</u>

373 voxels (5<1%) covering 1<1% of PostCG l (Postcentral Gyrus Left) with center at (-42,-26,+54)
345 voxels (46%) covering 8% of PreCG l (Precentral Gyrus Left) with center at (-44,-12,+52)
24 voxels (3%) covering <1% of not-labeled with center at (-34,-20,+54)

<u>Right cluster: x: +34, y: -24, z: +52; size: 550 voxel</u>

392 voxels (71%) covering 9% of PreCG r (Precentral Gyrus Right) with center at (+42,-14,+52)
124 voxels (23%) covering 4% of PostCG r (Postcentral Gyrus Right) with center at (+36,-26,+54)
34 voxels (6%) covering <1% of not-labeled with center at (+34,-16,+50)

Post-hoc seed based connectivity (SBC) analysis

Left fc-MVPA cluster as Seed

<u>Cluster 1: +50: , y: -18, z: -12; size: 491 voxel (correlation)</u>

251 voxels (51%) covering 18% of pMTG r (Middle Temporal Gyrus, posterior division Right) with center at (+52,-26,-8)
92 voxels (19%) covering 22% of pSTG r (Superior Temporal Gyrus, posterior division Right) with center at (+52,-22,-4)
22 voxels (4%) covering 2% of toMTG r (Middle Temporal Gyrus, temporooccipital part Right) with center at (+56,-40,+2)
3 voxels (1%) covering 1% of PT r (Planum Temporale Right) with center at (+44,-30,+10)
1 voxels (<1%) covering <1% of aSTG r (Superior Temporal Gyrus, anterior division Right) with center at (+52,-6,-12)
1 voxels (<1%) covering <1% of pSMG r (Supramarginal Gyrus, posterior division Right) with center at (+64,-40,+6)
121 voxels (25%) covering <1% of not-labeled with center at (+46,-24,-4)

<u>Cluster 2: x: +08 , y: -20, z: +58; size: 208 voxel (correlation)</u>

81 voxels (39%) covering 2% of PreCG r (Precentral Gyrus Right) with center at (+8,-22,+58)
46 voxels (22%) covering 1% of PreCG l (Precentral Gyrus Left) with center at (-8,-24,+60)
8 voxels (4%) covering <1% of PostCG r (Postcentral Gyrus Right) with center at (+10,-34,+56)
73 voxels (35%) covering <1% of not-labeled with center at (+10,-26,+58)

<u>Cluster 3: x: +18, y: +46, z: +26; size: 201 voxel (anti-correlation)</u>

79 voxels (39%) covering 1% of FP r (Frontal Pole Right) with center at (+22,+54,+14)
1 voxels (<1%) covering <1% of PaCiG r (Paracingulate Gyrus Right) with center at (+14,+36,+24)
121 voxels (6<1%) covering <1% of not-labeled with center at (+18,+48,+16)

Right fc-MVPA cluster as Seed

<u>Cluster 1: x: -52, y: -32, z: -04; size: 419 voxel (correlation)</u>

146 voxels (35%) covering 11% of pMTG l (Middle Temporal Gyrus, posterior division Left) with center at (-54,-36,-4)
84 voxels (2<1%) covering 1<1% of toMTG l (Middle Temporal Gyrus, temporooccipital part Left) with center at (-54,-50,+0)
80 voxels (19%) covering 2<1% of pSTG l (Superior Temporal Gyrus, posterior division Left) with center at (-62,-28,+2)
109 voxels (26%) covering <1% of not-labeled with center at (-54,-32,+0)

<u>Cluster 2: x: +18, y: +54, z: +04; size: 318 voxel (anti-correlation)</u>

135 voxels (42%) covering 2% of FP r (Frontal Pole Right) with center at (+24,+56,+4)
183 voxels (58%) covering <1% of not-labeled with center at (+18,+52,+4)

Overlap with canonical Large-Scale Brain Networks

Table S2: Title: Cluster overlap with canonical networks – group model (SP vs. GC)

Analysis	Cluster	MNI coordinates	Networks						
	Peak label	x, y, z	Fronto-parietal	Default	Limbic	Visual	Somato-sensory	Salience	Dorsal attention
Fc-MVPA	PostCG l	-36, -26, 50	0	0	0	0	536	18	38
	PreCG r	34, -24, 52	0	0	0	0	346	48	15
SBC (left seed)	pMTG r	50, -18, -12	0	344	0	0	64	0	0
	PreCG r	08, -20. -58	0	0	0	0	119	4	0
	FP r	18, 46, 26	57	14	0	0	0	1	0
SBC (right seed)	pMTG l	-52, -32, -04	22	257	0	0	40	7	3
	FP r	18, 54, 04	121	2	0	0	0	0	0

Overlap with canonical networks according to (Yeo et al., 2011). MNI: Montral Neurological Institute, PostCG l: Post central Gyrus right, PreCG r: Precentral gyrus right, pMTG r: post middle temporal gyrus right, pMTG l: post middle temporal gyrus left

'Pre vs. post' treatment model

fc-MVPA results

<u>Cluster 1: x: +26, y: -82, z: -14; size: 149 voxel</u>

125 voxels (84%) covering 14% of OFusG r (Occipital Fusiform Gyrus Right) with center at (+24,-80,-10)
6 voxels (4%) covering <1% of Cereb1 r (Cerebelum Crus1 Right) with center at (+18,-86,-20)
18 voxels (12%) covering <1% of not-labeled with center at (+24,-80,-2)

Post-hoc seed based connectivity (SBC) analysis

<u>Cluster 1: x: +26, y: -96 , z: +14; size: 1150 voxel (correlation)</u>

564 voxels (49%) covering 23% of OP r (Occipital Pole Right) with center at (+28,-94,+10)
241 voxels (21%) covering 12% of iLOC r (Lateral Occipital Cortex, inferior division Right) with center at (+38,-86,-4)
87 voxels (8%) covering 2% of sLOC r (Lateral Occipital Cortex, superior division Right) with center at (+30,-86,+16)
17 voxels (1%) covering 2% of OFusG r (Occipital Fusiform Gyrus Right) with center at (+30,-84,-16)
241 voxels (21%) covering <1% of not-labeled with center at (+28,-86,+4)

<u>Cluster 2: x: +28, y: -68, z: -22; size: 317 voxel (correlation)</u>

189 voxels (6<1%) covering 12% of Cereb6 r (Cerebelum 6 Right) with center at (+30,-66,-22)
46 voxels (15%) covering 6% of TOFusC r (Temporal Occipital Fusiform Cortex Right) with center at (+34,-56,-20)
45 voxels (14%) covering 2% of Cereb1 r (Cerebelum Crus1 Right) with center at (+40,-68,-24)
36 voxels (11%) covering 4% of OFusG r (Occipital Fusiform Gyrus Right) with center at (+34,-68,-18)
1 voxels (<1%) covering <1% of not-labeled with center at (+38,-70,-20)

<u>Cluster 3: x: -28, y: -82, z: 00; size: 208 voxel (correlation)</u>

85 voxels (41%) covering 4% of iLOC l (Lateral Occipital Cortex, inferior division Left) with center at (-32,-86,+2)
22 voxels (11%) covering 1% of OP l (Occipital Pole Left) with center at (-28,-92,+6)
4 voxels (2%) covering <1% of sLOC l (Lateral Occipital Cortex, superior division Left) with center at (-32,-86,+8)
97 voxels (47%) covering <1% of not-labeled with center at (-28,-84,+2)

Cluster 4: x: -28, y: -64, z: -18; 155 voxel (correlation)

101 voxels (65%) covering 8% of Cereb6 l (Cerebelum 6 Left) with center at (-26,-64,-20)
27 voxels (17%) covering 3% of OFusG l (Occipital Fusiform Gyrus Left) with center at (-28,-66,-18)
25 voxels (16%) covering 4% of TOFusC l (Temporal Occipital Fusiform Cortex Left) with center at (-30,-60,-18)
2 voxels (1%) covering <1% of Cereb45 l (Cerebelum 4 5 Left) with center at (-22,-50,-22)

Post hoc seed based connectivity (SBC) analysis - anticorrelations

Cluster 1: x: +44, y: -50 , z: +58; size: 328 voxel (anti-correlation)

200 voxels (61%) covering 14% of AG r (Angular Gyrus Right) with center at (+48,-52,+50)
59 voxels (18%) covering 1% of sLOC r (Lateral Occipital Cortex, superior division Right) with center at (+44,-58,+54)
42 voxels (13%) covering 3% of SPL r (Superior Parietal Lobule Right) with center at (+42,-50,+56)
2 voxels (1%) covering <1% of pSMG r (Supramarginal Gyrus, posterior division Right) with center at (+56,-46,+40)
25 voxels (8%) covering <1% of not-labeled with center at (+46,-56,+58)

Cluster 2: x: +30, y: +52, z: -18; size: 283 voxel (anti-correlation)

239 voxels (84%) covering 3% of FP r (Frontal Pole Right) with center at (+26,+54,-12)
44 voxels (16%) covering <1% of not-labeled with center at (+26,+52,-20)

Cluster 3: x: +08, y: -64, z: +44; size: 250 voxel (anti-correlation)

241 voxels (96%) covering 4% of Precuneous (Precuneous Cortex) with center at (+4,-64,+44)
9 voxels (4%) covering <1% of not-labeled with center at (+14,-58,+42)

Cluster 4: x: +04, y: +36, z: +36; size: 206 voxel (anti-correlation)

142 voxels (69%) covering 1<1% of PaCiG r (Paracingulate Gyrus Right) with center at (+4,+38,+32)
30 voxels (15%) covering 1% of SFG r (Superior Frontal Gyrus Right) with center at (+6,+38,+40)
2 voxels (1%) covering <1% of PaCiG l (Paracingulate Gyrus Left) with center at (-2,+38,+34)
32 voxels (16%) covering <1% of not-labeled with center at (+0,+38,+34)

Visualization of the result clusters including the subcortical and cerebellar regions

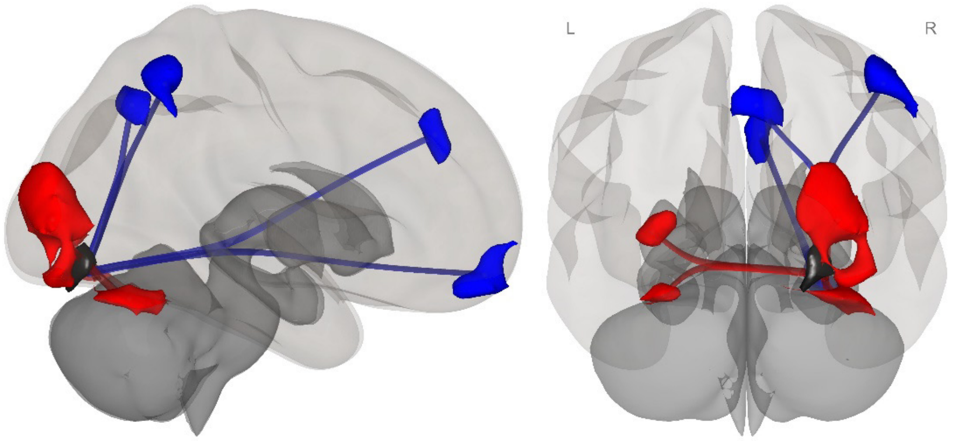

Figure S7: Seed based connectivity (SBC) maps of the 'pre > post' treatment model. presented on a semi inflated white matter template including subcortical structures and the cerebellum. Black shape: Seed region (right fusiform gyrus, occipital part); red shapes: positive correlations; blue connections: anticorrelations. Red connections: higher correlations in pre compared to post treatment condition; blue lines: higher anticorrelation in pre compared to post treatment condition.

Overlap with canonical Networks within analysis (pre-post)

Table S3: Cluster overlap with canonical networks – 'pre vs. post' treatment model

Analysis	Cluster	MNI coordinates	Networks						
	Peak label	x, y, z	Fronto-parietal	Default	Limbic	Visual	Somato-sensory	salience	Dorsal attention
Fc-MVPA	OFusG r	26, -82, -14	0	10	0	113	0	0	0
SBC positive	OP r	26, -96, +14	0	0	0	903	0	0	0
	Cereb6 r	28, -68, -22	103	4	1	89	0	119	1
	ILOC l	-28, -82, 00	0	0	0	107	0	0	0
	Cereb6 l	-28, -64, -18	3	0	0	35	0	110	2
SBC negative	AG r	44, -50, 58	262	35	0	0	0	0	30
	FP r	30, 52, -18	163	1	45	0	0	0	0
	Prec r	08, -64, 44	103	121	0	0	0	0	5
	PaCig r	04, 36, 36	102	86	0	0	0	0	0

Overlap with canonical networks according to (Yeo et al., 2011). OP r: Occipital pole right, Cereb6 r: Cerebellum 6right, ILOC l: Lateral occipital cortex inferior division left, Cereb6 l: Cerebellum 6 left, AG r: Angular gyrus right, FP r: Frontal pole right, Prec r: Precuneous cortex right, PaCig r.: paracingulate gyrus right

Requested additional analyses

Methods, Results and Discussion

Treatment response rate and connectivity strength

At the request of one reviewer, we calculated the treatment response rate (> 50% reduction in FSQ) and tested the difference in connectivity strength (in this case, the average deviation of connectivity from zero) between responders and non-responders. The response rate was 49.3%. There was no difference in connectivity between the groups. As discussed in the limitations section of the manuscript, we assume that clinical scores do not necessarily reflect brain function of basic cognitive processes. Consequently, the observed alterations in connectivity do not directly predict treatment response as measured by the FSQ.

With regard to the effectiveness of the one-session treatment, we would like to point out that, at post-measurement, 80.5% of SP participants showed a reduction of at least 30% in the FSQ.

Sensitivity analysis: excluding SP participants with comorbidity

At the request of one of the reviewers, additional sensitivity analyses should be conducted, excluding the SP participants with comorbidity (n = 9), in order to test the stability of effects when ruling out a potential impact of comorbidity. As it would be very time consuming to recalculate the models if individuals were to be removed from the analyses, we have created new regressors. One contains only SP participants without comorbidity, and the other contains SP participants with comorbidity. The latter was specified as a regressor of no interest. Second-level comparisons were then recalculated in both the 'SP vs. CG' and 'pre- vs. post' models.

The fc-MVPA results of the 'SP vs. CG' model show slightly smaller clusters than those in the main analysis (see Figure S8A and S8C). On the right, two clusters can be seen that merged into one large cluster in the main analysis. The cluster on the left hemisphere is significantly smaller than in the main analysis. It should be noted that the peak coordinates are similar to those of the main analysis, and all clusters are localized in the pre- and postcentral cortices. The effect size maps also reveal a comparable pattern of connectivity Figure S8B and S8D .

The fc-MVPA results for the 'pre vs. post' model are comparable with those of the main analysis (Figure S9A). The cluster is located in the right occipital fusiform gyrus, and the effect size maps are similar to those of the main analysis (Figure S9B).

Discussion: Excluding SP participants with comorbidity has no significant impact on the results. The smaller cluster size is probably due to reduced statistical power. It is possible that comorbid disorders that are more severe (such as severe depression, mania or psychosis) have a greater influence, but these were excluded from our study in advance.

Visualization of the fc-MVPA result clusters and effect size maps

Sensitivity Analysis 'SP vs. CG' model (N = 145)

fc-MVPA results

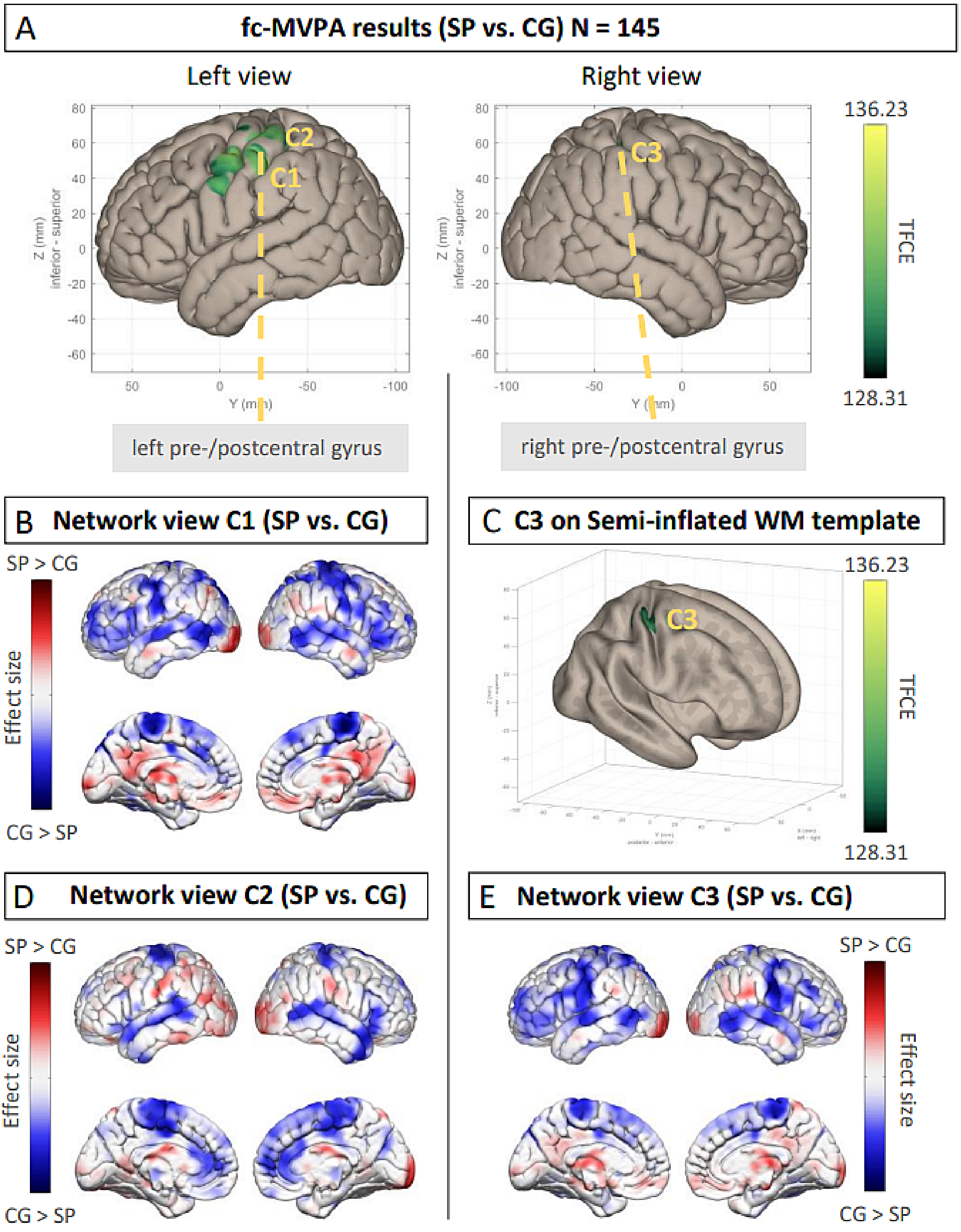

Figure S8: Sensitivity analysis of the group contrast 'SP vs. CG', excluding SP subjects with comorbidity (N = 9). (A) Fc-MVPA result clusters are shown in a left and right view and of a grey matter MNI template. (C) As cluster C3 is located deep within the sulcus, it is also displayed on a semi-inflated white matter template. (B, D and E) show the effect size maps of connectivity. SP: Spider Phobia group; CG: Control group; TFCE: Threshold Free Cluster Enhancement.

Detailed description of result clusters

Sensitivity Analysis ‘SP vs. CG’ model (N = 145)

fc-MVPA results

Left Cluster C1: x: -36 y: -26 z: +54; size 371 voxel

291 voxels (78%) covering 8% of PostCG l (Postcentral Gyrus Left) with center at (-42,-26,+54)
78 voxels (21%) covering 2% of PreCG l (Precentral Gyrus Left) with center at (-34,-22,+58)
2 voxels (1%) covering 0% of not-labeled with center at (-34,-24,+42)

Left Cluster C2: x: -46 y: -08 z: +50; size 160 voxel

153 voxels (96%) covering 4% of PreCG l (Precentral Gyrus Left) with center at (-50,-4,+46)
2 voxels (1%) covering 0% of MidFG l (Middle Frontal Gyrus Left) with center at (-36,+0,+46)
5 voxels (3%) covering 0% of not-labeled with center at (-58,+4,+40)

Right Cluster C3: x: +34 y: -26 z: +50; size 159 voxel

88 voxels (55%) covering 3% of PostCG r (Postcentral Gyrus Right) with center at (+34,-30,+56)
64 voxels (40%) covering 1% of PreCG r (Precentral Gyrus Right) with center at (+32,-24,+54)
7 voxels (4%) covering 0% of not-labeled with center at (+32,-24,+48)

Visualization of the fc-MVPA result clusters and effect size maps

Sensitivity Analysis 'pre vs. post' model (N = 63)

fc-MVPA results

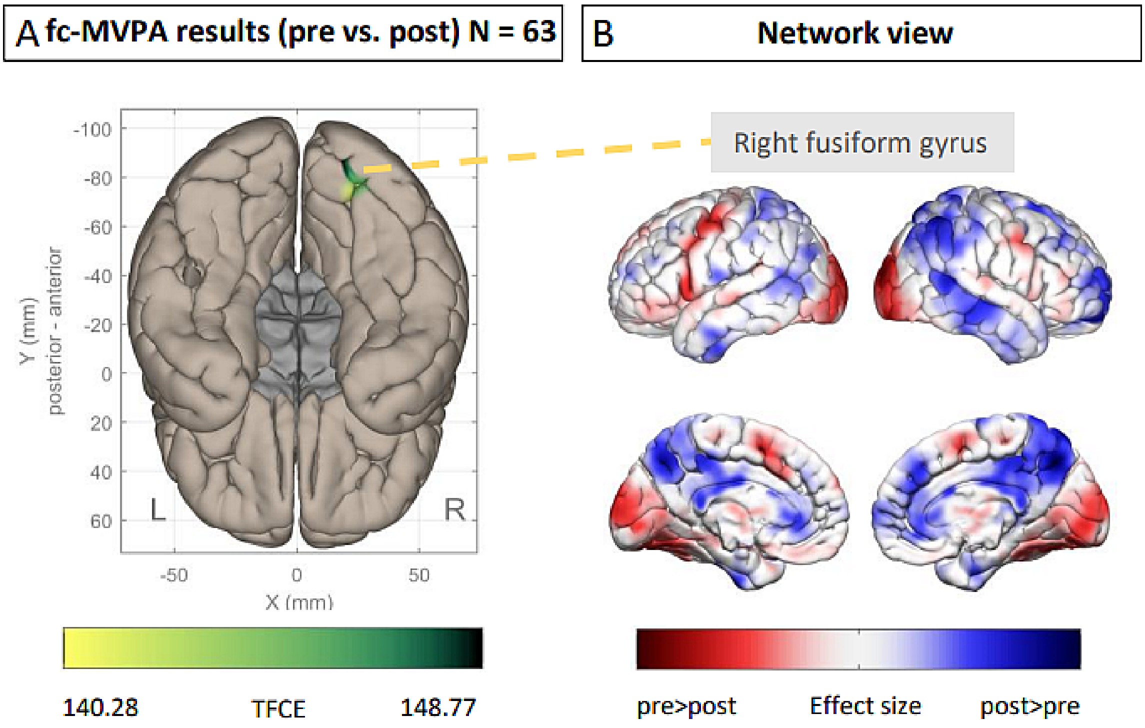

Figure S9: Sensitivity analysis of the within contrast 'pre vs. post', excluding SP subjects with comorbidity (N = 9). (A) Fc-MVPA result cluster is shown on an inferior view and of a grey matter MNI template. (B) Effect size maps of connectivity. TFCE: Threshold Free Cluster Enhancement.

Detailed description of result clusters

Sensitivity Analysis 'pre vs. post' model (N = 63)

fc-MVPA results

<u>Cluster 1: x: +24 y: -78 z: -10; size 121 voxel</u>

106 voxels (88%) covering 12% of OFusG r (Occipital Fusiform Gyrus Right) with center at (+24,-80,-8)

15 voxels (12%) covering 0% of not-labeled with center at (+26,-80,-4)

Sensitivity analysis: including SP participants without post-measurement

At the request of one reviewer, additional fc-MVPA analyses were performed, including 10 SP participants for whom there is no "post-measurement" because they withdrew from the study before therapy or before the second fMRI measurement. This increased the sample size to 164. As in the main analysis of the group contrast, an N:k ratio of 1:10 was chosen, resulting in 16 analyzed eigenpatterns. The integration of the additional 10 individuals significantly increased the sensitivity of the fc-MVPA, resulting in an overall connectivity pattern of 18 clusters in which the SP group

differed from the CG group. The clusters identified largely correspond to the regions identified by the main fc-MVPA and post hoc SBC analyses in the 'SP vs. CG' and 'pre vs. post' contrasts, which were used to determine the direction of the effects and the strength of the connections between these regions.

Overall, however, the extent of the clusters in the sensitivity analysis covers more regions than in the main analyses. For example, in addition to the visual cortices, the connectivity pattern extends along the temporal pathway to the amygdala. Parts of other subcortical structures, such as the hippocampus or nucleus accumbens, were also identified. See below for the TSFC maps (Figure S8) and cluster descriptions.

One reason for the differences between the main and sensitivity analyses may be greater power, another may be the higher symptom severity of the SP participants. The individuals who prematurely dropped out of the analysis have higher FAS values and lower BAT values (Table S4). In particular, the connectivity patterns within the additional regions identified, such as the hippocampus, amygdala and nucleus accumbens, could provide valuable starting points for predicting treatment success in future studies.

Table S4: Descriptive comparison of clinical data (questionnaire scores) of SP participants in the main analysis and sensitivity analysis for baseline measurement (pre-treatment).

	**SP in the main Analysis (N = 72)**	**Additional SP in the sensitivity analysis (N = 10)**
FSQ	78.93 (12.19)	87.80 (12.38)
BAT	6.10 (2.02)	5.60 (2.83)

SP: Spider Phobia group; FSQ: Fear of Spiders Questionnaire; BAT: Behavioral Avoidance Test

Visualization of the fc-MVPA result clusters

Sensitivity Analysis 'SP vs. CG' model (N = 164)

fc-MVPA results

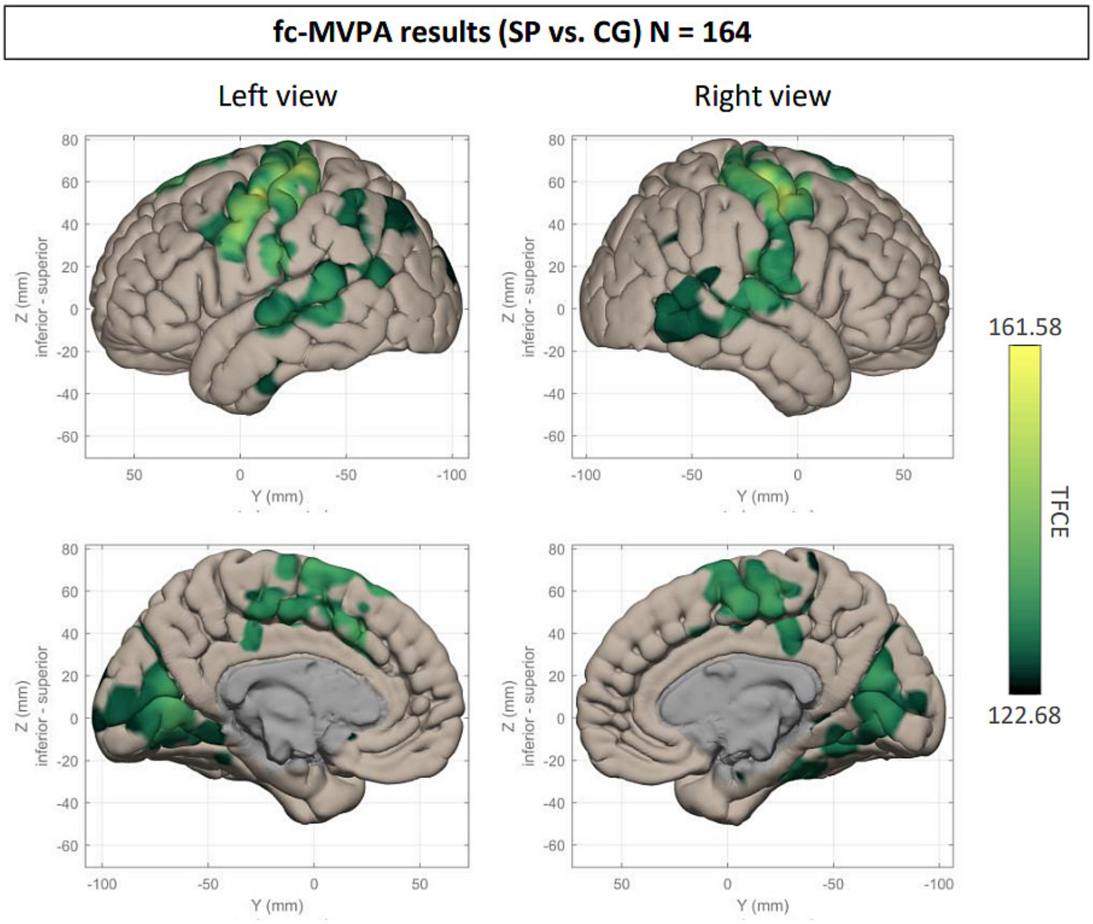

Figure S10: Sensitivity analysis of the group contrast 'SP vs. CG' including SP subjects (N=10) who withdrew from the study before the post measurement. Clusters are shown on a left and right view (top row) and a left medial and right medial view (bottom row) of an MNI grey matter template. SP: Spider Phobia group; CG: Control group; TFCE: Threshold Free Cluster Enhancement.

Detailed description of result clusters

Sensitivity Analysis 'SP vs. CG' model (N = 164)

fc-MVPA results

<u>Cluster 1: x: -34 y: -26 z: +52; size 9807 voxel</u>

1557 voxels (16%) covering 36% of PreCG l (Precentral Gyrus Left) with center at (-36,-14,+56)
1465 voxels (15%) covering 34% of PreCG r (Precentral Gyrus Right) with center at (+34,-14,+56)
1314 voxels (13%) covering 36% of PostCG l (Postcentral Gyrus Left) with center at (-44,-24,+50)
1056 voxels (11%) covering 33% of PostCG r (Postcentral Gyrus Right) with center at (+44,-22,+48)
385 voxels (4%) covering 14% of SFG l (Superior Frontal Gyrus Left) with center at (-10,+18,+64)
275 voxels (3%) covering 31% of CO r (Central Opercular Cortex Right) with center at (+54,-14,+14)
273 voxels (3%) covering 38% of SMA r (Juxtapositional Lobule Cortex -formerly Supplementary Motor Cortex- Right) with center at (+6,-4,+58)
210 voxels (2%) covering 8% of SFG r (Superior Frontal Gyrus Right) with center at (+14,+14,+64)
184 voxels (2%) covering 14% of pMTG r (Middle Temporal Gyrus, posterior division Right) with center at (+56,-28,-4)

173 voxels (2%) covering 27% of SMA L(Juxtapositional Lobule Cortex -formerly Supplementary Motor Cortex- Left) with center at (-4,-6,+56)
173 voxels (2%) covering 39% of PT r (Planum Temporale Right) with center at (+54,-22,+10)
127 voxels (1%) covering 3<1% of pSTG r (Superior Temporal Gyrus, posterior division Right) with center at (+56,-26,+2)
112 voxels (1%) covering 4% of MidFG l (Middle Frontal Gyrus Left) with center at (-44,+6,+44)
107 voxels (1%) covering 8% of PaCiG l (Paracingulate Gyrus Left) with center at (-8,+18,+40)
101 voxels (1%) covering 1<1% of CO l (Central Opercular Cortex Left) with center at (-46,-18,+18)
93 voxels (1%) covering 3% of MidFG r (Middle Frontal Gyrus Right) with center at (+42,+4,+56)
76 voxels (1%) covering 27% of HG r (Heschl's Gyrus Right) with center at (+50,-20,+10)
66 voxels (1%) covering 3% of PC (Cingulate Gyrus, posterior division) with center at (+2,-28,+40)
64 voxels (1%) covering 11% of PO l (Parietal Operculum Cortex Left) with center at (-42,-28,+18)
45 voxels (<1%) covering 3% of IC r (Insular Cortex Right) with center at (+38,-10,+12)
42 voxels (<1%) covering 3% of pSMG r (Supramarginal Gyrus, posterior division Right) with center at (+64,-40,+14)
42 voxels (<1%) covering 8% of PO r (Parietal Operculum Cortex Right) with center at (+44,-22,+16)
38 voxels (<1%) covering 3% of IC l (Insular Cortex Left) with center at (-36,-16,+14)
29 voxels (<1%) covering 1% of AC (Cingulate Gyrus, anterior division) with center at (-4,-6,+44)
24 voxels (<1%) covering 2% of SPL r (Superior Parietal Lobule Right) with center at (+26,-42,+58)
24 voxels (<1%) covering 8% of HG l (Heschl's Gyrus Left) with center at (-38,-24,+10)
20 voxels (<1%) covering 2% of toMTG r (Middle Temporal Gyrus, temporooccipital part Right) with center at (+58,-38,+2)
19 voxels (<1%) covering 3% of PT l (Planum Temporale Left) with center at (-40,-30,+12)
13 voxels (<1%) covering 1% of PaCiG r (Paracingulate Gyrus Right) with center at (+10,+14,+44)
4 voxels (<1%) covering <1% of aSMG r (Supramarginal Gyrus, anterior division Right) with center at (+52,-22,+32)
3 voxels (<1%) covering <1% of AG r (Angular Gyrus Right) with center at (+66,-46,+14)
2 voxels (<1%) covering <1% of aSMG l (Supramarginal Gyrus, anterior division Left) with center at (-58,-26,+22)
2 voxels (<1%) covering 1% of PP r (Planum Polare Right) with center at (+42,-20,-4)
1 voxels (<1%) covering <1% of SPL l (Superior Parietal Lobule Left) with center at (-34,-38,+52)
1688 voxels (17%) covering <1% of not-labeled with center at (+12,-8,+42)

Cluster 2: x: -54 y: -26 z: -04; size 1934 voxel

433 voxels (22%) covering 31% of pMTG l (Middle Temporal Gyrus, posterior division Left) with center at (-58,-28,-6)
303 voxels (16%) covering 32% of AG l (Angular Gyrus Left) with center at (-50,-56,+32)
230 voxels (12%) covering 5% of sLOC l (Lateral Occipital Cortex, superior division Left) with center at (-42,-70,+34)
224 voxels (12%) covering 57% of pSTG l (Superior Temporal Gyrus, posterior division Left) with center at (-62,-30,+0)
142 voxels (7%) covering 13% of pSMG l (Supramarginal Gyrus, posterior division Left) with center at (-56,-48,+22)
40 voxels (2%) covering 4% of pITG l (Inferior Temporal Gyrus, posterior division Left) with center at (-52,-18,-26)
37 voxels (2%) covering 4% of toMTG l (Middle Temporal Gyrus, temporooccipital part Left) with center at (-58,-44,+4)
13 voxels (1%) covering 2% of PT l (Planum Temporale Left) with center at (-58,-34,+10)
8 voxels (<1%) covering 2% of aITG l (Inferior Temporal Gyrus, anterior division Left) with center at (-58,-10,-36)
8 voxels (<1%) covering <1% of iLOC l (Lateral Occipital Cortex, inferior division Left) with center at (-48,-64,+14)
4 voxels (<1%) covering 1% of aSTG l (Superior Temporal Gyrus, anterior division Left) with center at (-62,-10,-4)
1 voxels (<1%) covering <1% of aMTG l (Middle Temporal Gyrus, anterior division Left) with center at (-62,-12,-8)
1 voxels (<1%) covering <1% of pTFusC l (Temporal Fusiform Cortex, posterior division Left) with center at (-42,-20,-22)
490 voxels (25%) covering <1% of not-labeled with center at (-54,-30,+0)

Cluster 3: x: -06 y: -62 z: -04; size 3206 voxel

526 voxels (16%) covering 35% of LG l (Lingual Gyrus Left) with center at (-10,-68,-4)
365 voxels (11%) covering 21% of LG r (Lingual Gyrus Right) with center at (+10,-64,-8)
226 voxels (7%) covering 25% of Cereb45 l (Cerebelum 4 5 Left) with center at (-12,-50,-16)
181 voxels (6%) covering 24% of ICC r (Intracalcarine Cortex Right) with center at (+8,-70,+12)
164 voxels (5%) covering 11% of Cereb6 r (Cerebelum 6 Right) with center at (+22,-54,-22)
163 voxels (5%) covering 25% of ICC l (Intracalcarine Cortex Left) with center at (-4,-76,+8)
159 voxels (5%) covering 23% of Hippocampus r with center at (+24,-14,-20)
122 voxels (4%) covering 2<1% of Cereb45 r (Cerebelum 4 5 Right) with center at (+16,-48,-18)
103 voxels (3%) covering 14% of pTFusC r (Temporal Fusiform Cortex, posterior division Right) with center at (+34,-32,-22)
103 voxels (3%) covering 4% of OP l (Occipital Pole Left) with center at (-6,-96,+2)
97 voxels (3%) covering 12% of TOFusC r (Temporal Occipital Fusiform Cortex Right) with center at (+30,-48,-14)
91 voxels (3%) covering 14% of Cuneal r (Cuneal Cortex Right) with center at (+8,-74,+24)

86 voxels (3%) covering 2% of sLOC l (Lateral Occipital Cortex, superior division Left) with center at (-20,-78,+36)
66 voxels (2%) covering 13% of Cuneal l (Cuneal Cortex Left) with center at (-12,-74,+26)
52 voxels (2%) covering 15% of Amygdala r with center at (+24,-6,-18)
44 voxels (1%) covering 13% of Ver6 (Vermis 6) with center at (+2,-68,-8)
43 voxels (1%) covering 1% of Precuneous (Precuneous Cortex) with center at (-12,-72,+26)
43 voxels (1%) covering 7% of Ver45 (Vermis 4 5) with center at (+0,-50,-12)
34 voxels (1%) covering 3% of Cereb6 l (Cerebelum 6 Left) with center at (-28,-50,-26)
33 voxels (1%) covering 23% of SCC r (Supracalcarine Cortex Right) with center at (+2,-74,+14)
16 voxels (<1%) covering 1% of OP r (Occipital Pole Right) with center at (+2,-92,+6)
15 voxels (<1%) covering 5% of pPaHC r (Parahippocampal Gyrus, posterior division Right) with center at (+30,-26,-22)
13 voxels (<1%) covering 18% of SCC l (Supracalcarine Cortex Left) with center at (-2,-76,+16)
9 voxels (<1%) covering <1% of PC (Cingulate Gyrus, posterior division) with center at (+18,-44,+2)
8 voxels (<1%) covering 1% of aPaHC r (Parahippocampal Gyrus, anterior division Right) with center at (+30,-20,-24)
6 voxels (<1%) covering 1% of OFusG l (Occipital Fusiform Gyrus Left) with center at (-24,-66,-6)
3 voxels (<1%) covering <1% of Hippocampus l with center at (-22,-40,-4)
2 voxels (<1%) covering <1% of pTFusC l (Temporal Fusiform Cortex, posterior division Left) with center at (-26,-44,-20)
2 voxels (<1%) covering <1% of TOFusC l (Temporal Occipital Fusiform Cortex Left) with center at (-26,-46,-20)
431 voxels (13%) covering <1% of not-labeled with center at (+0,-66,+2)

Cluster 4: x: +60 y: -66 z: -06; size 481 voxel

296 voxels (62%) covering 25% of toMTG r (Middle Temporal Gyrus, temporooccipital part Right) with center at (+62,-52,-2)
83 voxels (17%) covering 4% of iLOC r (Lateral Occipital Cortex, inferior division Right) with center at (+56,-64,-2)
44 voxels (9%) covering 6% of toITG r (Inferior Temporal Gyrus, temporooccipital part Right) with center at (+52,-54,-10)
58 voxels (12%) covering <1% of not-labeled with center at (+66,-56,+0)

Cluster 5: x: -14 y: -96 z: +18; size 126 voxel

117 voxels (93%) covering 4% of OP l (Occipital Pole Left) with center at (-14,-96,+18)
9 voxels (7%) covering <1% of not-labeled with center at (-14,-90,+18)

Cluster 6: x: +20 y: -78 z: +34; size 63 voxel

27 voxels (43%) covering 1% of sLOC r (Lateral Occipital Cortex, superior division Right) with center at (+18,-80,+38)
12 voxels (19%) covering 2% of Cuneal r (Cuneal Cortex Right) with center at (+18,-78,+34)
7 voxels (11%) covering <1% of Precuneous (Precuneous Cortex) with center at (+16,-76,+40)
17 voxels (27%) covering <1% of not-labeled with center at (+18,-78,+36)

Cluster 7: x: -26 y: +26 z: -08; size 39 voxel

7 voxels (18%) covering <1% of FOrb l (Frontal Orbital Cortex Left) with center at (-28,+28,-6)
32 voxels (82%) covering <1% of not-labeled with center at (-24,+28,-4)

Cluster 8: x: -02 y: +08 z: -04; size 44 voxel

13 voxels (3<1%) covering 12% of Accumbens l with center at (-8,+12,-4)
11 voxels (25%) covering 1% of SubCalC (Subcallosal Cortex) with center at (-4,+12,-4)
2 voxels (5%) covering <1% of Caudate l with center at (-10,+14,-2)
18 voxels (41%) covering <1% of not-labeled with center at (-4,+8,-2)

Cluster 9: x: +10 y: -40 z: +70; size 37 voxel

37 voxels (10<1%) covering 1% of PostCG r (Postcentral Gyrus Right) with center at (+8,-40,+72)

Cluster 10: x: +30 y: -04 z: +26; size 9 voxel

9 voxels (10<1%) covering <1% of not-labeled with center at (+28,+0,+28)

Cluster 11: x: +10 y: +08 z: -08; size 46 voxel

16 voxels (35%) covering 19% of Accumbens r with center at (+8,+10,-6)
6 voxels (13%) covering 1% of Caudate r with center at (+8,+8,-2)
24 voxels (52%) covering <1% of not-labeled with center at (+8,+4,-2)

Cluster 12: x: +46 y: -48 z: +22; size 11 voxel

8 voxels (73%) covering 1% of AG r (Angular Gyrus Right) with center at (+46,-48,+24)
3 voxels (27%) covering <1% of not-labeled with center at (+42,-46,+24)

Cluster 13: x: +50 y: -56 z: +30; size 19 voxel

19 voxels (10<1%) covering 1% of AG r (Angular Gyrus Right) with center at (+48,-56,+32)

Cluster 14: x: -62 y: -20 z: +38; size 3 voxel

3 voxels (10<1%) covering <1% of not-labeled with center at (-62,-18,-38)

Cluster 15: x: -20 y: -48 z: -36; size 6 voxel

6 voxels (10<1%) covering <1% of not-labeled with center at (-20,-46,-36)

Cluster 16: x: +02 y: +10 z: +06; size 6 voxel

6 voxels (10<1%) covering <1% of not-labeled with center at (+2,+10,+6)

Cluster 17: x: -46 y: -12 z: -02; size 8 voxel

6 voxels (75%) covering 2% of HG l (Heschl's Gyrus Left) with center at (-48,-12,+2)
1 voxels (13%) covering <1% of PP l (Planum Polare Left) with center at (-46,-12,-2)
1 voxels (13%) covering <1% of not-labeled with center at (-46,-10,+2)

Cluster 18: x: +16 y: -68 z: +42; size 18 voxel

15 voxels (83%) covering <1% of Precuneous (Precuneous Cortex) with center at (+14,-68,+46)
2 voxels (11%) covering <1% of sLOC r (Lateral Occipital Cortex, superior division Right) with center at (+16,-72,+46)
1 voxels (6%) covering <1% of not-labeled with center at (+14,-64,+46)

Supplementary Discussion

On request, we added a brief discussion of the differences between our findings and the results of task-based studies that have examined anxiety disorders and the effect of therapeutic interventions. Earlier task based studies and meta-analyses have indicated the occurrence of treatment-related alterations of functional activity in frontal regions, such as the anterior cingulate cortex and the anterior insular regions, during the presentation of threatening or emotional stimuli (e.g. Picó-Pérez et al., 2023; Schrammen et al., 2022). It is hypothesised that these results primarily reflect altered salience allocation to these stimuli. However, in the resting state, there is no constant salience allocation to stimuli or tasks, which ultimately explains the differences between these studies and our results.

References

Andersson, J. L. R., Hutton, C., Ashburner, J., Turner, R., & Friston, K. (2001). Modeling Geometric Deformations in EPI Time Series. *NeuroImage*, *13*(5), 903–919. https://doi.org/10.1006/nimg.2001.0746

Ashburner, J. (2007). A fast diffeomorphic image registration algorithm. *NeuroImage*, *38*(1), 95–113. https://doi.org/10.1016/j.neuroimage.2007.07.007

Ashburner, J., & Friston, K. J. (2005). Unified segmentation. *NeuroImage*, *26*(3), 839–851. https://doi.org/10.1016/j.neuroimage.2005.02.018

Behzadi, Y., Restom, K., Liau, J., & Liu, T. T. (2007). A component based noise correction method (CompCor) for BOLD and perfusion based fMRI. *NeuroImage*, *37*(1), 90–101. https://doi.org/10.1016/j.neuroimage.2007.04.042

Calhoun, V. D., Wager, T. D., Krishnan, A., Rosch, K. S., Seymour, K. E., Nebel, M. B., Mostofsky, S. H., Nyalakanai, P., & Kiehl, K. (2017). The impact of T1 versus EPI spatial normalization templates for fMRI data analyses. *Human Brain Mapping*, *38*(11), 5331–5342. https://doi.org/10.1002/hbm.23737

Chai, X. J., Castañón, A. N., Öngür, D., & Whitfield-Gabrieli, S. (2012). Anticorrelations in resting state networks without global signal regression. *NeuroImage*, *59*(2), 1420–1428. https://doi.org/10.1016/j.neuroimage.2011.08.048

Choy, Y., Fyer, A. J., & Lipsitz, J. D. (2007). Treatment of specific phobia in adults. *Clinical Psychology Review*, *27*(3), 266–286. https://doi.org/10.1016/j.cpr.2006.10.002

Chumbley, J., Worsley, K., Flandin, G., & Friston, K. (2010). Topological FDR for neuroimaging. *NeuroImage*, *49*(4), 3057–3064. https://doi.org/10.1016/j.neuroimage.2009.10.090

Davis III, T. E., Ollendick, T. H., & Öst, L.-G. (Hrsg.). (2012). *Intensive one-session treatment of specific phobias* (S. xvi, 255). Springer Science + Business Media. https://doi.org/10.1007/978-1-4614-3253-1

Friston, K. J., Williams, S., Howard, R., Frackowiak, R. S. J., & Turner, R. (1996). Movement-Related effects in fMRI time-series. *Magnetic Resonance in Medicine*, *35*(3), 346–355. https://doi.org/10.1002/mrm.1910350312

Friston, Karl. J., Ashburner, J., Frith, C. D., Poline, J.-B., Heather, J. D., & Frackowiak, R. S. J. (1995). Spatial registration and normalization of images. *Human Brain Mapping*, *3*(3), 165–189. https://doi.org/10.1002/hbm.460030303

Hallquist, M. N., Hwang, K., & Luna, B. (2013). The nuisance of nuisance regression: Spectral misspecification in a common approach to resting-state fMRI preprocessing reintroduces noise and obscures functional connectivity. *NeuroImage*, *82*, 208–225. https://doi.org/10.1016/j.neuroimage.2013.05.116

Nieto-Castanon, A. (2020). *Handbook of functional connectivity Magnetic Resonance Imaging methods in CONN*. Hilbert Press.

Nieto-Castanon, A. (2022a). Brain-wide connectome inferences using functional connectivity MultiVariate Pattern Analyses (fc-MVPA). *PLOS Computational Biology*, *18*(11), e1010634. https://doi.org/10.1371/journal.pcbi.1010634

Nieto-Castanon, A. (2022b). *Preparing fMRI Data for Statistical Analysis* (arXiv:2210.13564). arXiv. https://doi.org/10.48550/arXiv.2210.13564

Nieto-Castanon, A., & Whitfield-Gabrieli, S. (2022). *CONN functional connectivity toolbox: RRID SCR_009550, release 22*. https://doi.org/10.56441/hilbertpress.2246.5840

Olatunji, B. O., & Deacon, B. (2008). Specificity of disgust sensitivity in the prediction of fear and disgust responding to a brief spider exposure. *Journal of Anxiety Disorders*, *22*(2), 328–336. https://doi.org/10.1016/j.janxdis.2007.03.007

Penny, W. D., Friston, K. J., Ashburner, J. T., Kiebel, S. J., & Nichols, T. E. (2011). *Statistical Parametric Mapping: The Analysis of Functional Brain Images*. Elsevier.

Picó-Pérez, M., Fullana, M. A., Albajes-Eizagirre, A., Vega, D., Marco-Pallarés, J., Vilar, A., Chamorro, J., Felmingham, K. L., Harrison, B. J., Radua, J., & Soriano-Mas, C. (2023). Neural predictors of cognitive-behavior therapy outcome in anxiety-related disorders: A meta-analysis of task-based fMRI studies. *Psychological Medicine*, *53*(8), 3387–3395. https://doi.org/10.1017/S0033291721005444

Power, J. D., Mitra, A., Laumann, T. O., Snyder, A. Z., Schlaggar, B. L., & Petersen, S. E. (2014). Methods to detect, characterize, and remove motion artifact in resting state fMRI. *NeuroImage*, *84*, 320–341. https://doi.org/10.1016/j.neuroimage.2013.08.048

Schrammen, E., Roesmann, K., Rosenbaum, D., Redlich, R., Harenbrock, J., Dannlowski, U., & Leehr, E. J. (2022). Functional neural changes associated with psychotherapy in anxiety disorders – A meta-analysis of longitudinal fMRI studies. *Neuroscience & Biobehavioral Reviews*, *142*, 104895. https://doi.org/10.1016/j.neubiorev.2022.104895

Smith, S. M., & Nichols, T. E. (2009). Threshold-free cluster enhancement: Addressing problems of smoothing, threshold dependence and localisation in cluster inference. *NeuroImage*, *44*(1), 83–98. https://doi.org/10.1016/j.neuroimage.2008.03.061

Szymanski, J., & O'Donohue, W. (1995). Fear of Spiders Questionnaire. *Journal of Behavior Therapy and Experimental Psychiatry*, *26*(1), 31–34. https://doi.org/10.1016/0005-7916(94)00072-t

Tejavibulya, L., Peterson, H., Greene, A., Gao, S., Rolison, M., Noble, S., & Scheinost, D. (2022). Large-scale differences in functional organization of left- and right-handed individuals using whole-brain, data-driven analysis of connectivity. *NeuroImage*, *252*, 119040. https://doi.org/10.1016/j.neuroimage.2022.119040

Whitfield-Gabrieli, S., & Nieto-Castanon, A. (2012). Conn: A functional connectivity toolbox for correlated and anticorrelated brain networks. *Brain Connectivity*, *2*(3), 125–141. https://doi.org/10.1089/brain.2012.0073

Whitfield-Gabrieli, S., Nieto-Castanon, A., & Ghosh, S. (2011). *Artifact Detection Tools (ART)* (Version 7(19)) [Software]. https://www.nitrc.org/projects/artifact_detect/

Wittchen, H.-U., & Pfister, H. (1997). *DIA-X-Interviews: Manual für Screening-Verfahren und Interview; Interviewheft*. Swets & Zeitlinger. https://hdl.handle.net/11858/00-001M-0000-000E-AAAF-C

Wolitzky-Taylor, K. B., Horowitz, J. D., Powers, M. B., & Telch, M. J. (2008). Psychological approaches in the treatment of specific phobias: A meta-analysis. *Clinical Psychology Review*, *28*(6), 1021–1037. https://doi.org/10.1016/j.cpr.2008.02.007

Worsley, K. J., Marrett, S., Neelin, P., Vandal, A. C., Friston, K. J., & Evans, A. C. (1996). A unified statistical approach for determining significant signals in images of cerebral activation. *Human Brain Mapping*, *4*(1), 58–73. https://doi.org/10.1002/(SICI)1097-0193(1996)4:1<58::AID-HBM4>3.0.CO;2-O

Yeo, B. T., Krienen, F. M., Sepulcre, J., Sabuncu, M. R., Lashkari, D., Hollinshead, M., Roffman, J. L., Smoller, J. W., Zöllei, L., Polimeni, J. R., Fischl, B., Liu, H., & Buckner, R. L. (2011). The organization of the human cerebral cortex estimated by intrinsic functional connectivity. *Journal of Neurophysiology*, *106*(3), 1125–1165. https://doi.org/10.1152/jn.00338.2011

Zhang, C., Cahill, N. D., Arbabshirani, M. R., White, T., Baum, S. A., & Michael, A. M. (2016). Sex and Age Effects of Functional Connectivity in Early Adulthood. *Brain Connectivity*, *6*(9), 700–713. https://doi.org/10.1089/brain.2016.0429

Zlomke, K., & Davis, T. E. (2008). One-session treatment of specific phobias: A detailed description and review of treatment efficacy. *Behavior Therapy*, *39*(3), 207–223. https://doi.org/10.1016/j.beth.2007.07.003
